# Effectiveness of a Suicide Prevention Module for Adults in Substance Use Disorder Treatment: A Stepped-Wedge Cluster-Randomized Clinical Trial

**DOI:** 10.1001/jamanetworkopen.2022.2945

**Published:** 2022-04-06

**Authors:** Richard K. Ries, Adam L. Livengood, David Huh, Amanda H. Kerbrat, Martina Fruhbauerova, Brianna Turner, Katherine Anne Comtois

**Affiliations:** 1 Department of Psychiatry & Behavioral Sciences, School of Medicine, University of Washington, Seattle; 2 Institute for Research and Education to Advance Community Health, Washington State University, Seattle; 3 Formerly with Department of Psychiatry & Behavioral Sciences, School of Medicine, University of Washington, Seattle, Washington; 4 School of Social Work, University of Washington, Seattle; 5 Department of Psychology, College of Arts and Sciences, University of Kentucky, Lexington; 6 Department of Psychology, University of Victoria, Victoria, Canada

## Abstract

**Importance:**

Individuals with substance use disorders (SUDs) are at high risk for suicide. The Preventing Addiction Related Suicide (PARS) module is the first suicide prevention module developed in and for community substance use intensive outpatient programs (IOPs).

**Objective:**

To evaluate the effectiveness of PARS on suicide-related outcomes (ie, knowledge, attitudes, and help-seeking behavior) compared with usual care.

**Design, Setting, and Participants:**

This stepped-wedge cluster-randomized clinical trial was conducted from 2017 to 2020, with follow-up assessments conducted after treatment and at 1, 3, and 6 months. Participants included adult outpatients in SUD treatment at community IOPs across western Washington state. Data were analyzed from July 1, 2020, to January 20, 2022.

**Interventions:**

The intervention, PARS, was a 1-session secondary prevention module administered by trained SUD counselors consisting of didactic presentations and group discussions about suicide risk factors, warning signs, and actions to take if suicide risk is observed in self or others. The control group received usual care.

**Main Outcomes and Measures:**

Primary outcomes were suicide knowledge, attitudes about suicide, and help-seeking behavior among patients enrolled in an IOP.

**Results:**

A total of 906 participants (mean [SD] age, 37.5 [12.0] years; 540 [59.6%] men) were included, with 478 participants receiving usual care and 428 participants receiving PARS. In intent-to-treat analysis from baseline to after treatment, there was a greater improvement in suicide knowledge (*d* = 0.15; 95% CI, 0.08 to 0.23; *P* < .001) and a greater reduction in maladaptive attitudes (*d* = 0.18; 95% CI, 0.14 to 0.25; *P* < .001) for PARS participants compared with those receiving usual care. Improvements were maintained at follow-up for suicide knowledge (1 month: *d* = 0.16; 95% CI, 0.07 to 0.22; *P* < .001; 3 months: *d* = 0.12; 95% CI, 0.05 to 0.19; *P* = .001; 6 months: *d* = 0.13; 95% CI, 0.06 to 0.20; *P* < .001) and reductions in maladaptive attitudes (1 month: *d* = 0.20; 95% CI, 0.12 to 0.23; *P* < .001; 3 months: *d* = 0.10; 95% CI, 0.05 to 0.16; *P* < .001; 6 months: *d* = 0.14; 95% CI, 0.09 to 0.19; *P* < .001), with 788 participants (87.0%) of the sample responding across time points. From baseline to 6 months, there was a greater improvement in help-seeking in the PARS group vs usual care (*d* = 0.16; 95% CI, 0.01 to 0.32; *P* = .04).

**Conclusions and Relevance:**

This stepped-wedge cluster-randomized clinical trial found that PARS was superior to usual care in improving suicide knowledge, maladaptive attitudes, and help-seeking in adults undergoing community addiction treatment. As a 1-session IOP module developed in partnership with community addiction agencies, PARS has the potential for wide impact in the national suicide prevention strategy.

**Trial Registration:**

ClinicalTrials.gov Identifier: NCT03166709

## Key Points

**Question:**

Does a secondary prevention module in community addiction treatment improve patients’ suicide knowledge, attitudes, and help-seeking compared with usual care?

**Findings:**

In this stepped-wedge cluster-randomized clinical trial including 906 participants at 15 treatment sites, the Preventing Addiction Related Suicide (PARS) prevention module produced consistently greater improvements compared with usual care in suicide knowledge and a greater reduction in maladaptive attitudes about suicide across all time points, and greater improvement in help-seeking by 6 months.

**Meaning:**

These findings suggest that PARS was effective in improving suicide prevention outcomes and has the potential for wide dissemination and implementation.

## Teaser

This stepped-wedge cluster-randomized clinical trial evaluates the effectiveness of a suicide prevention module integrated into addiction treatment on suicide-related outcomes.

## Introduction

Suicide remains a serious public health issue, with numerous studies reporting that most suicides are related to mental disorders. Less well known is that the risk of suicide is 10-fold higher for individuals with alcohol use disorder,^1,2,3,4^ 14-fold higher for individuals injecting drugs, and 17-fold higher for individuals who use multiple different drugs,^3,4^ although there has been relatively little focus on suicide prevention interventions within addiction treatment settings. Approximately 2.5 million individuals a year receive substance use disorder (SUD) treatment in the United States.^5^ These individuals report a high prevalence of prior suicidal thoughts^6,7,8,9^ and suicide attempts.^6,7,8,9,10,11,12,13,14,15,16,17,18,19^ In prospective studies, receiving treatment (a proxy for severity) also is associated with increased suicide attempts.^6,9,10,12,20,21^ SUD treatment may present unique opportunities to prevent suicide, since treatment and recovery staff engage a high-risk population that is both concentrated and accessible.

**Figure 1.**
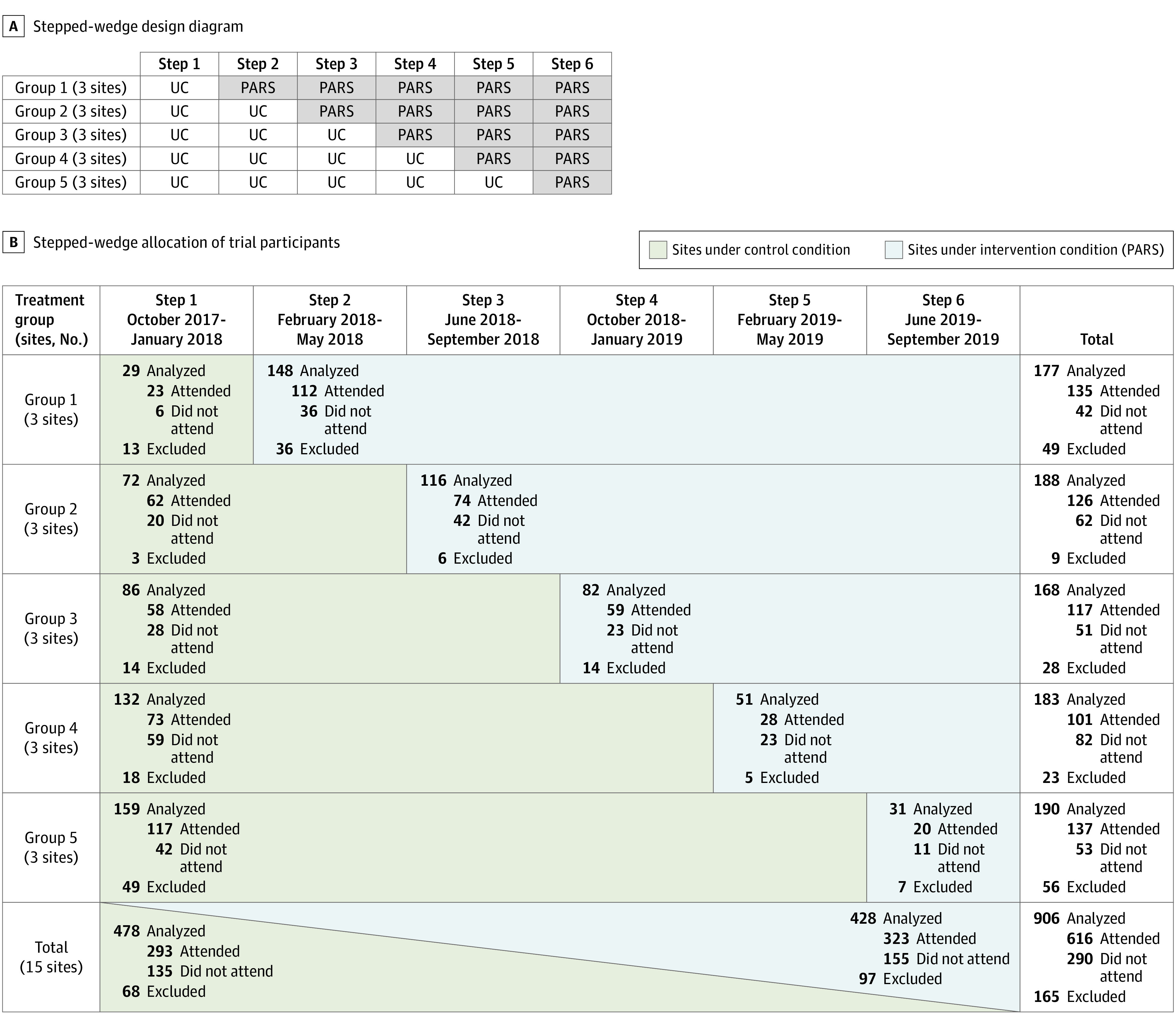
Cluster and Participant Recruitment Flowcharts A, This stepped-wedge effectiveness trial randomly assigned 15 community IOPs into 5 groups of 3 sites each. The 5 groups were randomly ordered to implement the Preventing Addiction Related Suicide (PARS) program (shaded cells) at one of the subsequent 5 study steps (ie, steps 2-6), with each step lasting 4 months. At step 1, all were in the usual care (UC) condition (unshaded cells). Once beginning PARS, sites continued to administer PARS through the end of step 6. B, All 1071 participants in selected intensive outpatient programs were recruited; 906 participants were randomized and 165 were excluded, including 157 who did not consent, 6 who did not meet study criteria, and 2 who either did not provide data or withdrew consent. Specification of *attended* indicates the participant attended study session (UC or PARS) and were included in the as-treated secondary analysis.

Responding to these issues, we developed Preventing Addiction Related Suicide (PARS),^22^ an interactive psychoeducational module developed and tested within partner SUD intensive outpatient programs (IOPs), the most common form of community addiction treatment.^23^ In our pilot study,^22^ PARS showed excellent effectiveness and feasibility along with significant changes in patients’ suicide knowledge, maladaptive attitudes, and help-seeking behavior. The goal of this study was to replicate pilot results using a fully powered stepped-wedge cluster-randomized clinical trial (SW-CRT) of PARS vs usual care in 15 community SUD treatment sites (Figure 1A). This pragmatic SW-CRT design was chosen in partnership with SUD IOPs, for which workload and lack of resources made individual and within-site cluster randomization infeasible. The SW-CRT with IOP sites as clusters facilitated rollout of PARS to all partner sites, provided equity in the order PARS training was received, and prevented contamination. We hypothesized that, among IOP patients, PARS would result in the following suicide-related outcomes: increased accurate knowledge, reduced maladaptive attitudes, and increased help-seeking.

## Methods

This stepped-wedge cluster-randomized clinical trial was approved by an institutional review board at the University of Washington, and a data and safety monitoring board monitored for adverse events throughout the trial. All participants provided written informed consent. Primary and secondary outcomes were predefined and prespecified, including how and when they were assessed. The trial protocol and statistical analysis plan are included in Supplement 1. This study is reported per the Consolidated Standards of Reporting Trials extension for the SW-CRT (SW-CRT CONSORT Extension).

### Setting

Recruitment was conducted between October 11, 2017, and September 16, 2019, at 15 IOP sites from 4 SUD treatment agencies in western Washington state. For each site, 1 or 2 IOPs were selected to participate in the study, and patients in these groups were recruited in each 16-week step. Participants completed baseline, posttreatment, and 1-, 3-, and 6-month follow-up assessments. Final follow-up was May 28, 2020.

### Participants

All adult English-speaking patients with an SUD enrolled in participating IOPs were recruited to participate. The exclusion criterion was any concern regarding safe and voluntary participation (eg, psychosis, custody conflict). No participants were excluded on this basis. Patients were told separately by agency and research staff that the study was voluntary, their participation would not affect their treatment, and their data would not be shared with the agency unless they reported suicide risk. A week ahead of recruitment, counselors informed patients about the study via printed flyers containing study details. On the day of recruitment, informed consent was conducted by research staff in a group format. Interested participants provided written consent then completed baseline measures on tablets or paper questionnaires. Figure 1B summarizes the flow of participants through the study.

### Measures

The PARS Suicide Knowledge Scale is an 11-item measure adapted from the Staff Suicide Prevention Survey^24^ assessing factual understanding of suicide and closely mapped to the content of PARS. Correct responses were summed to create a score from 0 to 11, with higher scores indicating more accurate knowledge. Previous research demonstrated validity before and after suicide training,^24,25^ and test-retest reliability from baseline to after treatment in the usual care condition ranged from 0.46 to 0.62.

The PARS Attitude Scale, also adapted from the Staff Suicide Prevention Survey,^24^ consists of 6 items evaluating attitudes about suicide stigma and prevention. Responses on a 5-point scale from 1, indicating strongly disagree to 5 indicating strongly agree were summed to create a score from 6 to 30, with higher score indicating more maladaptive attitudes about suicide. Internal consistency based on the pilot sample^22^ was α = .73 to .97 and in this full sample was α = .59 to .73.

The PARS Help-Seeking Scale consists of 4 items assessing help-seeking behavior in the context of suicidal thoughts or feelings (eg, calling a crisis/suicide hotline) developed for our pilot trial^22^ based on prior suicide prevention programs.^25^ Participants reported the frequency of past-month help-seeking on behalf of self or others, including friends and family members, from 0, indicating never, to 4, indicating more than 3 times. Responses were summed to create a score from 0 to 16, with higher scores indicating more help-seeking behavior. Internal consistency based on the pilot sample^22^ was α = .82 to .87 and in this full sample was α = .52 to .76.

The Alcohol, Smoking and Substance Involvement Screening Test^26^ assesses frequency of substance use (lifetime and past 3 months). The Suicidal Behavior Questionnaire-Revised (SBQ-R) is a valid and reliable 4-item self-report assessment of suicidal thoughts and behaviors^27,28,29^ with a nonclinical cutoff of 7 for elevated suicide risk.^28^ Recent alcohol use was measured by the number of days in the last 30 days that participants used alcohol. Demographics, including race and ethnicity, were asked based on NIH definitions, further specified with “check all that apply” (to correctly determine multiracial identity), Latinx and Asian ethnicities by nationality, and an other option for race and gender. These were recoded according to NIH guidelines. Race and ethnicity were assessed to characterize the sample.

The PARS Adherence Rating Scale assessed the fidelity of counselors’ initial delivery of PARS with respect to empathy, positivity, interactivity, responsiveness to questions, content competency, outline completion, and an overall rating. Ratings had 5 anchors: poor, mediocre, acceptable, good, and excellent, with 3 or higher on the Overall rating indicating fidelity to PARS. This scale was based on Motivational Interviewing fidelity measurement.^30,31^

The PARS Counselor Acceptability Scale consists of 12 items assessing acceptability and feasibility of PARS. Responses on a 5-point scale from 1, indicating strongly disagree, to 5, indicating strongly agree were summed for a score from 12 to 60, with higher scores indicating more acceptability.

### Study Interventions

#### Control: Usual Care

Although IOPs require at least monthly individual counseling sessions, the primary modality is a group format, typically including 6 to 12 patients who attend 3 sessions per week. Each 2- to 3-hour group session includes both didactic information and process discussion.^32^ IOPs have approximately 24 sessions, with new patients entering at any session.

Prior to recruitment, agency leadership selected a particular IOP session to be their usual care study session (ie, the session that would be replaced by PARS in the study design). To minimize usual care variability, the study session was one focused on grief, depression, or coping with negative emotions. Counselors continued to provide this study session at the same point in the IOP series (eg, session 8) as long as their site was randomized to usual care. Further fidelity to usual care was not assessed. Other treatment (eg, mental health, pharmacotherapy) was provided as usual. As Washington state mandates referral to self-help groups as part of IOPs, this also occurred as usual.

#### Intervention: PARS

PARS is a single 2- to 3-hour suicide prevention module designed to replace one IOP group session. PARS is organized by slides in presentation software guiding a combination of didactic presentations and group discussions. Topics include the strong link between addiction and suicide, suicide myths and facts, common triggers of suicidal thoughts and behaviors, suicide risk and protective factors, warning signs of suicide, the overlap of overdose and suicide,^33,34^ how to prevent addiction-related suicide, and creating a safety card.

The developer of PARS (R.K.R.) provided training to counselors in a single 2- to 3-hour session using the same presentation slide content and process used with patients. To maximize the value to our community partners and assure a counselor trained in PARS was available when needed, all counselors were offered PARS training. Training occurred 2 weeks prior to the first PARS study session, after which the usual care study session was replaced by PARS and counselors were instructed to provide PARS for the study session through the end of the study. The initial delivery of PARS at each site was observed by an adherence coder. IOP sites determined that direct observation would be less disruptive to patients than recording the sessions.

### Procedures

As shown in Figure 1, this SW-CRT involved a sequential crossover of groups of IOP sites (ie, groups of clusters) from the control (usual care) to intervention (PARS) arm, so that every group began in the control condition and eventually received the intervention. Randomization was conducted prior to study start by a study methodologist (D.H.) uninvolved in participant enrollment and blinded to the identity of the study sites via preassigned site codes. The sites were block randomized into 5 groups of 3 sites and then randomly ordered. All 15 sites started as usual care in step 1, and participants received the treatment (usual care or PARS) to which their site was randomized during the step in which they were recruited. To meet the goal of 10 clients enrolled per site per step, 1 or 2 IOPs for each site were selected to be the focus of this study based on number of groups offered and group size. Given that patients attended an IOP for 8 to 12 weeks and each step was 16 weeks, each step recruited new patients.

Within each step, recruitment, consent, and baseline assessment occurred approximately 1 week before the study session, to maximize the chance that participants would receive the study intervention. All patients in the selected IOPs were approached to participate. A brief posttreatment assessment of knowledge and attitudes was conducted 1 week after the study session, with longitudinal assessment of knowledge, attitudes, and help-seeking behavior at 1, 3, and 6 months.

Follow-up was conducted via a Research Electronic Data Capture (REDCap)^35,36^ online survey, sent by text or email based on participant preference. Follow-up surveys were conducted by phone, if requested. Phone assessors were not blinded to condition, as outcomes were measured via questionnaires and no items required assessor judgment. Participants were reimbursed with a $30 gift card for each assessment, except a brief posttreatment assessment, which was $20. To minimize attrition, participants were offered an additional incentive of $20 for completing 2 outcome assessments or $30 for completing all 3.

### Statistical Analysis

#### Sample Size Calculation

A sample size of 900 participants across 15 sites was targeted for the study based on the minimum number of participants necessary to detect a 12.5–percentage point difference in any help-seeking behavior between PARS and usual care, assuming a 15.6% base rate of helping behavior observed in a pilot study^22^ and 80% statistical power. We assumed 10 participants recruited per site (ie, 5 groups of 3 sites each) in each of the 6 steps, an intraclass correlation of 0.05 to account for the greater similarity of participants within site, and 25% fewer participants, owing to attrition or underrecruitment.

#### Data Analyses

To evaluate the effect of PARS vs usual care on participant outcomes, we used generalized linear mixed modeling to account for the clustered design of the study. All study participants who were randomized and completed a baseline assessment were included in the primary outcome analyses (ie, an intent-to-treat approach). The primary outcomes were: suicide knowledge, maladaptive attitudes about suicide, and help-seeking behavior. Each outcome variable was regressed on study step, treatment (PARS = 1; usual care = 0), time, and the treatment-by-time interaction in separate models. The time variable was divided into 4 planned contrasts: after treatment vs baseline, 1 month vs baseline, 3 months vs baseline, and 6 months vs baseline. With participants nested within 15 sites and 5 longitudinal assessments per participant, we included site- and participant-specific intercepts to account for the repeated measures design and the greater similarity of participants from the same site.

As planned, we also conducted secondary outcome analyses to evaluate the effect of PARS vs usual care among participants who were exposed to the intervention. These as-treated intervention outcome analyses included only participants who attended the study session, usual care or PARS, depending on the allocation of each participant. We adjusted for multiple statistical tests using the Benjamini and Hochberg approach,^37^ assuming a false discovery rate of 0.10 across all primary and secondary analyses.

Analyses were conducted with R version 4.1.2 (R Project for Statistical Computing). *P* values were 2-sided, and statistical significance was set at α = .05. Data were analyzed from July 1, 2020, to January 20, 2022.

## Results

### Participants

The final intent-to-treat sample included 906 participants, with age ranging from 18 to 78 years (mean [SD] age, 37.5 [12.0] years), and 540 participants (59.6%) were men. Demographic and clinical characteristics of the intent-to-treat and as-treated samples (ie, those who attended the study session) are summarized in Table 1 and were generally comparable. All sites (ie, clusters) completed all steps and received the intervention as randomized. Figure 1B shows the flow of participants through the trial. Of the 1071 approached IOP patients, 165 were excluded: 157 did not consent to participate, 6 did not meet study criteria, 1 had to leave after consent and never provided data, and 1 withdrew consent.

**Table 1.** Baseline Characteristics of Intent-to-Treat and As-Treated Samples

Characteristic	All (N = 906)	Participants, No. (%)			
		Intent-to-treat sample (N = 906)		As-treated sample (n = 616)^a^	
		UC (n = 478)	PARS (n = 428)	UC (n = 323)	PARS (n = 293)
Attended study session					
No	290 (32.0)	155 (32.4)	135 (31.5)	NA	NA
Yes	616 (68.0)	323 (67.6)	293 (68.5)	323 (100)	293 (100)
Gender					
Men	540 (59.6)	269 (56.3)	271 (63.3)	192 (59.4)	191 (65.2)
Women	350 (38.6)	201 (42.1)	149 (34.8)	126 (39.0)	97 (33.1)
Transgender or nonconforming	4 (0.4)	2 (0.4)	2 (0.5)	2 (0.6)	0
Missing	12 (1.3)	6 (1.3)	6 (1.4)	3 (0.9)	5 (1.7)
Age, mean (SD), y^b^	37.5 (12.0)	37.3 (12.4)	37.7 (11.7)	37.3 (12.7)	37.8 (11.8)
Hispanic or Latinx ethnicity					
No	817 (90.2)	439 (91.8)	378 (88.3)	294 (91.0)	260 (88.7)
Yes	73 (8.1)	31 (6.5)	42 (9.8)	24 (7.4)	27 (9.2)
Missing	16 (1.8)	8 (1.7)	8 (1.9)	5 (1.5)	6 (2.0)
Race					
American Indian or Alaska Native	26 (2.9)	16 (3.3)	10 (2.3)	11 (3.4)	5 (1.7)
Asian	18 (2.0)	11 (2.3)	7 (1.6)	6 (1.9)	5 (1.7)
Black	33 (3.6)	16 (3.3)	17 (4.0)	6 (1.9)	11 (3.8)
Native Hawaiian or other Pacific Islander	8 (0.9)	6 (1.3)	2 (0.5)	4 (1.2)	2 (0.7)
White	673 (74.3)	347 (72.6)	326 (76.2)	238 (73.7)	229 (78.2)
Other^c^	26 (2.9)	13 (2.7)	13 (3.0)	11 (3.4)	7 (2.4)
>1 race	108 (11.9)	62 (13.0)	46 (10.7)	43 (13.3)	29 (9.9)
Missing	14 (1.5)	7 (1.5)	7 (1.6)	4 (1.2)	5 (1.7)
Marital status					
Never married	436 (48.1)	234 (49.0)	202 (47.2)	165 (51.1)	134 (45.7)
Married	240 (26.5)	123 (25.7)	117 (27.3)	80 (24.8)	87 (29.7)
Separated or divorced	198 (21.9	101 (21.1)	97 (22.7)	66 (20.4)	61 (20.8)
Widowed	12 (1.3)	7 (1.5)	5 (1.2)	4 (1.2)	4 (1.4)
Missing	20 (2.2)	13 (2.7)	7 (1.6)	8 (2.5)	7 (2.4)
Education					
<High school diploma or GED	88 (9.7)	65 (13.6)	23 (5.4)	38 (11.8)	18 (6.1)
High school diploma or GED	247 (27.3)	128 (26.8)	119 (27.8)	88 (27.2)	75 (25.6)
Some college or technical training^d^	383 (42.3)	197 (41.2)	186 (43.5)	125 (38.7)	136 (46.4)
Bachelor’s or graduate degree	170 (18.8)	79 (16.5)	91 (21.3)	66 (20.4)	59 (20.1)
Missing	18 (2.0)	9 (1.9)	9 (2.1)	6 (1.9)	5 (1.7)
Employment					
Employed	520 (57.4)	257 (53.8)	263 (61.4)	177 (54.8)	183 (62.5)
Disabled or retired	65 (7.2)	43 (9.0)	22 (5.1)	27 (8.4)	9 (3.1)
Unemployed^e^	276 (30.5)	151 (31.6)	125 (29.2)	104 (32.2)	89 (30.4)
Missing	45 (5.0)	27 (5.6)	18 (4.2)	15 (4.6)	12 (4.1)
Elevated lifetime suicide risk^f^	423 (46.7)	211 (44.1)	212 (49.5)	138 (42.7)	147 (50.2)
Any use (lifetime)					
Marijuana^g^	847 (93.5)	448 (93.7)	399 (93.2)	298 (92.3)	273 (93.2)
Stimulants^h^	734 (81.0)	390 (81.6)	344 (80.4)	259 (80.2)	235 (80.2)
Opioids^i^	636 (70.2)	329 (68.8)	307 (71.7)	209 (64.7)	206 (70.3)
Regular use (past 3 mo)^j^					
Marijuana^g^	240 (26.5)	123 (25.7)	117 (27.3)	79 (24.5)	83 (28.3)
Stimulants^h^	128 (14.1)	75 (15.7)	53 (12.4)	44 (13.6)	32 (10.9)
Opioids^i^	147 (16.2)	85 (17.8)	62 (14.5)	51 (15.8)	40 (13.7)
Alcohol use (past 30 d)^k^					
Any	281 (31.0)	132 (27.6)	149 (34.8)	103 (31.9)	106 (36.2)
Median (IQR)	4.0 (2.0-10.0)	4.0 (2.0-10.0)	5.0 (2.0-10.5)	4.0 (2.0-10.0)	5.0 (2.0-14.0)

Abbreviations: GED, General Equivalency Diploma; PARS, Preventing Addiction Related Suicide; UC, usual care.

^a^ The as-treated sample consists of the subset of participants who attended the PARS or UC study session.

^b^ Age was missing for 40 participants.

^c^ Other race includes 16 Mexican American or Chicano individuals, 5 other Hispanic or Latino individuals, 3 Puerto Rican individuals, and 2 individuals who did not specify.

^d^ Some college or technical training also includes associate’s degree.

^e^ Unemployed includes 14 participants receiving unemployment benefits and 262 participants not working and not receiving unemployment, disability, or retirement income.

^f^ Elevated risk was defined as reaching a nonclinical cutoff of 7 or higher on the Suicidal Behaviors Questionnaire–Revised. Data were missing for 10 participants.

^g^ Marijuana includes both recreational marijuana and medical marijuana. Data were missing for 4 participants for lifetime use and 6 participants for use in the past 3 months.

^h^ Stimulants include cocaine, methamphetamine, and prescription stimulants (eg, amphetamine and dextroamphetamine) if misused; missing for 8 individuals for lifetime use and 13 individuals for past 3 months use.

^i^ Opioids include street opioids (eg, heroin) and prescription opioids (eg, hydrocodone) if misused; missing for n = 6 (lifetime), n = 6 (past 3 months).

^j^ Regular substance use (past 3 months) denotes participants who reported using weekly or daily or almost daily.

^k^ Past 30-day alcohol use missing for 38 participants.

### PARS

Raters found all PARS counselors adherent on the PARS Adherence Rating Scale. Following the 3-hour PARS training, counselors rated PARS highly acceptable on the PARS Counselor Acceptability Scale (mean [SD] score, 55.4 [5.0] points).

#### Missing Data

With respect to the primary study outcomes, 713 participants (78.7%) had outcome data from all 5 assessments, and 193 participants (21.3%) were missing 1 or more assessments. Since the rate of missing data was comparable across treatment conditions and the generalized linear mixed modeling approach uses all available information from participants, missing data should not bias outcome analyses.

#### Descriptive Data on Study Outcomes

Table 2 provides descriptive statistics on outcomes by time point and condition. At baseline, mean (SD) suicide knowledge scores were 7.42 (1.68) points among usual care participants and 7.76 (1.70) points among PARS participants, mean (SD) maladaptive attitudes scores were 12.81 (3.25) points among usual care participants and 12.58 (3.38) points among PARS participants, and mean (SD) help-seeking scores were 0.63 (1.45) points among usual care participants and 0.48 (1.14) points among PARS participants. Both PARS and usual care participants improved in the knowledge of suicide facts the most after treatment, and scores remained stable across all subsequent time points. However, compared with usual care participants, PARS participants’ mean (SD) scores improved more after treatment (7.66 [1.51] vs 8.53 [1.69]) and remained higher throughout follow-up (1 month: 7.59 [1.68] points vs 8.41 [1.78] points; 3 months: 7.53 [1.82] points vs 8.26 [1.77] points; 6 months: 7.47 [1.89] points vs 8.24 [1.83] points). Similarly, mean scores of maladaptive suicide-related attitudes decreased in both groups after treatment, although compared with the usual care group, the decrease in mean (SD) maladaptive attitude scores was steeper for PARS participants after treatment (12.68 [3.40] vs 11.09 [3.75]), and mean (SD) scores remained lower throughout follow-up (1 month: 12.52 [3.43] vs 11.12 [3.92]; 3 months: 12.15 [3.37] vs 11.37 [4.07]; 6 months, 12.29 [3.60] vs 11.14 [3.88]). Mean (SD) help-seeking scores improved for the usual care group at 1 month (0.71 [1.74]) and 3 months (0.73 [2.03]) but declined at 6 months (0.57 [1.54]). In contrast, mean (SD) help-seeking scores declined for the PARS group at 1 month (0.39 [0.96]) and improved at 3 months (0.51 [1.45]) and 6 months (0.53 [1.66]). No study-related harms or adverse events were reported.

**Table 2.** Descriptive Summaries of Primary Outcomes by Time Point and Condition

Time point	Suicide knowledge^a^		Maladaptive attitudes^b^		Help-seeking^c^	
	UC (n = 478)	PARS (n = 428)	UC (n = 478)	PARS (n = 428)	UC (n = 478)	PARS (n = 428)
Baseline						
Mean (SD)	7.42 (1.68)	7.76 (1.70)	12.81 (3.25)	12.58 (3.38)	0.63 (1.45)	0.48 (1.14)
Median (IQR)	8 (7-9)	8 (7-9)	13 (10-15)	12 (10-15)	0 (0-1)	0 (0-0)
Missing, No. (%)	0 (0.0)	0 (0.0)	9 (1.9)	9 (2.1)	4 (0.8)	6 (1.4)
After treatment						
Mean (SD)	7.66 (1.51)	8.53 (1.69)	12.68 (3.40)	11.09 (3.75)	NA	NA
Median (IQR)	8 (7-9)	9 (8-10)	13 (10-15)	11 (8-13)	NA	NA
Missing, No. (%)	72 (15.1)	52 (12.1)	77 (16.1)	58 (13.6)	NA	NA
1-mo follow-up						
Mean (SD)	7.59 (1.68)	8.41 (1.78)	12.52 (3.43)	11.12 (3.92)	0.71 (1.74)	0.39 (0.96)
Median (IQR)	8 (7-9)	9 (8-10)	12.5 (10-15)	11 (8-13)	0 (0-1)	0 (0-0)
Missing, No. (%)	60 (12.6)	41 (9.6)	70 (14.6)	47 (11.0)	68 (14.2)	48 (11.2)
3-mo follow-up						
Mean (SD)	7.53 (1.82)	8.26 (1.77)	12.15 (3.37)	11.37 (4.07)	0.73 (2.03)	0.51 (1.45)
Median (IQR)	8 (7-9)	9 (8-9)	12 (10-14)	11 (8-14)	0 (0-0)	0 (0-0)
Missing, No. (%)	74 (15.5)	40 (9.3)	94 (19.7)	46 (10.7)	80 (16.7)	43 (10.0)
6-mo follow-up						
Mean (SD)	7.47 (1.89)	8.24 (1.83)	12.29 (3.60)	11.14 (3.88)	0.57 (1.54)	0.53 (1.66)
Median (IQR)	8 (7-9)	9 (8-9)	12 (10-15)	11 (8-14)	0 (0-0)	0 (0-0)
Missing, No. (%)	58 (12.1)	37 (8.6)	75 (15.7)	46 (10.7)	67 (14.0)	40 (9.3)

Abbreviations: PARS, Preventing Addiction Related Suicide; UC, usual care.

^a^ Assessed by 11-item PARS Suicide Knowledge Scale; scored as number of correct responses, ranging from 0 to 11; higher scores indicate more accurate suicide knowledge.

^b^ Agreement with maladaptive attitudes assessed by 6-item PARS Attitude Scale; scoring sums responses on 5-point scale, ranging from 6 to 30; lower scores indicate less agreement with maladaptive attitudes.

^c^ Frequency of help-seeking for self or on behalf of others assessed by 4-item PARS Help-Seeking Scale; scoring sums responses on 5-point scale, ranging from 0 to 16; higher scores indicate more help-seeking behavior.

#### Intent-to-Treat Intervention Outcome Analyses

**Figure 2.**
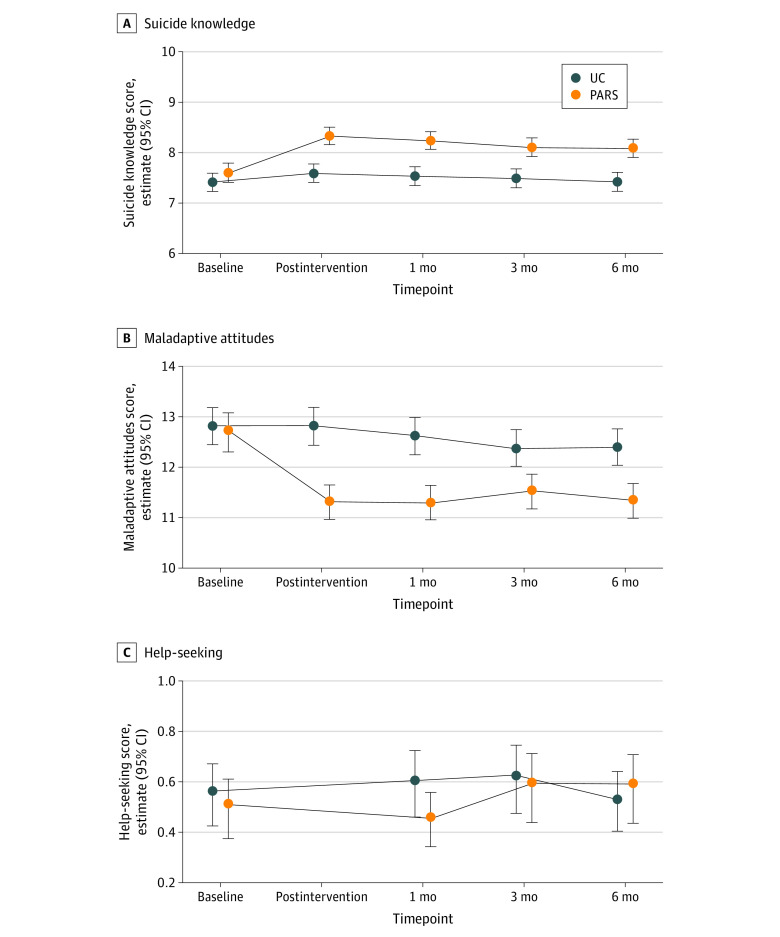
Predicted Outcomes at Baseline, After Treatment, and 1-, 3-, and 6-Month Follow-up by Treatment From the Intent-to-Treat Analysis Nonoverlapping confidence intervals correspond with a statistically significant difference at *P* < .05. PARS indicates Preventing Addiction Related Suicide; UC, usual care.

Figure 2 summarizes the predicted mean suicide knowledge, maladaptive attitudes, and help-seeking from the intent-to-treat outcome analysis by time point and condition. From baseline to after treatment in the PARS group compared with the usual care group, there was a greater improvement in suicide knowledge (estimate = 0.6; 95% CI, 0.3 to 0.8; *P* < .001) and a greater reduction in maladaptive attitudes (estimate = −1.4; 95% CI, −1.8 to −1.02; *P* < .001). Improvements in suicide knowledge (0.4- to 0.5-point greater improvement between 1 and 6 months) and reductions in maladaptive attitudes (0.7- to 1.2-point greater reduction between 1 and 6 months) were maintained at follow-up (Table 3). From baseline to 6 months, there was greater improvement (estimate = 0.16; 95% CI, 0.01 to 0.32; *P* = .04) in help-seeking in PARS vs usual care.

**Table 3.** Standardized Intervention Effects From Intent-to-Treat and As-Treated Analyses After Treatment and at 1, 3, and 6 Months^a^

Outcome	After treatment		1 Month		3 Months		6 Months	
	*d* (95% CI)	*P* value	*d* (95% CI)	*P* value	*d* (95% CI)	*P* value	*d* (95% CI)	*P* value
Intent-to-treat analysis								
Suicide knowledge^b^	0.15 (0.08 to 0.23)	<.001	0.16 (0.07 to 0.22)	<.001	0.12 (0.05 to 0.19)	.001	0.13 (0.06 to 0.20)	<.001
Maladaptive attitudes^c^	0.18 (0.14 to 0.25)	<.001	0.20 (0.12 to 0.23)	<.001	0.10 (0.05 to 0.16)	<.001	0.14 (0.09 to 0.19)	<.001
Help-seeking^d^	NA	NA	−0.14 (−0.30 to 0.02)	.08	0.04 (−0.11 to 0.19)	.65	0.16 (0.01 to 0.32)	.04
As-treated analysis								
Suicide knowledge^b^	0.23 (0.16 to 0.34)	<.001	0.25 (0.15 to 0.32)	<.001	0.16 (0.07 to 0.25)	.001	0.15 (0.07 to 0.24)	<.001
Maladaptive attitudes^c^	0.26 (0.20 to 0.33)	<.001	0.26 (0.20 to 0.32)	<.001	0.19 (0.13 to 0.26)	<.001	0.19 (0.12 to 0.25)	<.001
Help-seeking^d^	NA	NA	−0.08 (−0.27 to 0.15)	.47	−0.04 (−0.24 to 0.15)	.67	0.14 (−0.06 to 0.34)	.16

^a^ Positive results are in the direction of improvement. Effect size estimates control for baseline levels of the corresponding outcome (ie, 6 months vs baseline, 3 months vs baseline, 1 month vs baseline, and after treatment vs baseline).

^b^ Assessed by 11-item Preventing Addiction Related Suicide Suicide Knowledge Scale; scored as number of correct responses, ranging from 0 to 11; higher scores indicate more accurate suicide knowledge.

^c^ Agreement with maladaptive attitudes assessed by 6-item Preventing Addiction Related Suicide Attitude Scale; scoring sums responses on 5-point scale, ranging from 6 to 30; lower scores indicate less agreement with maladaptive attitudes.

^d^ Frequency of help-seeking for self or on behalf of others assessed by 4-item Preventing Addiction Related Suicide Help-Seeking Scale; scoring sums responses on 5-point scale, ranging from 0 to 16; higher scores indicate more help-seeking behavior.

Table 3 summarizes standardized intervention effect sizes. From baseline to after treatment, there was a greater improvement in suicide knowledge (*d* = 0.15; 95% CI, 0.08 to 0.23; *P* < .001) and a greater reduction in maladaptive attitudes (*d* = 0.18; (95% CI, 0.14 to 0.25; *P* < .001) for PARS participants compared with those receiving usual care. Improvements were maintained at follow-up for suicide knowledge (1 month: *d* = 0.16; 95% CI, 0.07 to 0.22; *P* < .001; 3 months: *d* = 0.12; 95% CI, 0.05 to 0.19; *P* = .001; 6 months: *d* = 0.13; 95% CI, 0.06 to 0.20; *P* < .001) and reductions in maladaptive attitudes (1 month: *d* = 0.20; 95% CI, 0.12 to 0.23; *P* < .001; 3 months: *d* = 0.10; 95% CI, 0.05 to 0.16; *P* < .001; 6 months: *d* = 0.14; 95% CI, 0.09 to 0.19; *P* < .001), corresponding with the threshold for a small effect size per Cohen.^38^

#### As-Treated Intervention Outcome Analyses

When the analyses were limited to participants who attended the PARS or corresponding usual care comparison sessions, the as-treated intervention effects with respect to suicide knowledge and maladaptive attitudes were generally larger, ranging from a 0.15- to 0.26-SD greater improvement for PARS over usual care, which correspond to small or small-to-medium effect sizes. With respect to help-seeking, the as-treated intervention effects differed from the intent-to-treat analysis in that PARS was not associated with a statistically significant improvement in help-seeking at 6 months, compared with usual care (Table 3).

## Discussion

To our knowledge, this is the first RCT of a suicide prevention intervention designed for, and tested in, community SUD treatment settings. Compared with usual care, participants assigned to the PARS condition showed significant improvements in their suicide knowledge and maladaptive attitudes at 1, 3, and 6 months. Increasing help-seeking has been recognized as a challenge in the suicide prevention field,^39^ and the effect of PARS on help-seeking was only significant at 6 months.

A goal of PARS from its inception was the development of an effective and feasible intervention that fit into SUD counselors’ usual workflow. Although working with a high suicide-risk population, most SUD counselors are not trained or licensed to give acute suicide treatment; however, PARS, as a secondary prevention intervention (as opposed to acute treatment), fits within SUD counselors’ scope of practice. Furthermore, by repeatedly delivering the PARS module in successive IOP groups, SUD counselors receive continued practice exposure to suicide prevention skills, which may translate into improved clinical care. Open discussion of experiences around suicide (a core component of PARS) may additionally help decrease stigma, a key issue in the recognition and intervention of suicidality.^40^

### Limitations

There were several limitations to this study. First, to maximize external validity, cluster randomization, limited training, and brief surveys were used in this trial. In particular, it would have been preferable to assess help-seeking throughout the follow-up period, rather than via past–30-day individual assessments at 3 follow-up time points over 6 months, and to know more about other health care utilization during this period. Second, prior to this study, Washington state mandated suicide prevention training for behavioral health workers, including community SUD counselors, possibly affecting counselors’ baseline suicide knowledge, and potentially restricting the range of improvement in patient outcomes. Furthermore, results are based on group IOPs and may not generalize to other types of SUD treatment.

## Conclusions

In this stepped-wedge cluster-randomized clinical trial, PARS was superior to usual care in improving suicide knowledge, maladaptive attitudes, and help-seeking in community IOPs. As substance-related suicide and overdose numbers increase and overlap, effective prevention interventions are needed in SUD treatment settings. With high acceptability and feasibility, PARS has the potential for wide impact.

## Notes

Supplement 1.
Trial Protocol and Statistical Analysis Plan

Supplement 2.
Data Sharing Statement.
