# Supplementary material for: Effectiveness of a Suicide Prevention Module for Adults in Substance Use Disorder Treatment: A Stepped-Wedge Cluster-Randomized Clinical Trial
Source: JAMA Netw Open. 2022 Apr 6;5(4):e222945. doi: 10.1001/jamanetworkopen.2022.2945 (PMC8987906; doi:10.1001/jamanetworkopen.2022.2945)
Supplement: Supplement 1. — Trial Protocol and Statistical Analysis Plan [file jamanetwopen-e222945-s001.pdf]

INSTRUCTIONS

- **If you are requesting a determination** about whether your activity is human subjects research or qualifies for exempt status, you may skip all questions except those marked with a ◯. For example (1.1) must be answered.
- **Answer all questions**. If a question is not applicable to your research or if you believe you have already answered a question elsewhere in the application, state "NA" (and if applicable, refer to the question where you provided the information). If you do not answer a question, the IRB does not know whether the question was overlooked or whether it is not applicable. This may result in unnecessary "back and forth" for clarification. Use non-technical language as much as possible.
- To check a box, place an "X" in the box. To fill in a text box, make sure your cursor is within the gray text box bar before typing or pasting text.
- The word "you" refers to the researcher and all members of the research team, unless otherwise specified.
- For collaborative research, describe only the information that is relevant to you unless you are requesting that the UW IRB provide the review and oversight for your collaborators as well.
- You may reference other documents (such as a grant application) if they provide the requested information in non-technical language. Be sure to provide the document name, page(s), and specific sections, and upload it to ***Zipline***. Also, describe any changes that may have occurred since the document was written (for example, changes that you've made during or after the grant review process). In some cases, you may need to provide additional details in the answer space as well as referencing a document.

INDEX

**1** Overview	**6** Children (Minors) and Parental Permission	**10** Risk / Benefit Assessment
**2** Participants	**7** Assent of Children (Minors)	**11** Economic Burden to Participants
**3** International Research Setting	**8** Consent of Adults	**12** Resources
**4** Recruiting and Screening Participants	**9** Privacy and Confidentiality	**13** Other Approvals, Permissions, and Regulatory Issues
**5** Procedures		

1 OVERVIEW

Study Title: Preventing Addiction Related Suicide (PARS) - Controlled Trial of Secondary Suicide Prevention

1.1 Home institution. Identify the home institution of the lead researcher as listed on the IRB application. Provide any helpful explanatory information.

In general, the home institution is the institution (1) that provides the researcher's paycheck and that considers him/her to be a paid employee, or (2) at which the researcher is a matriculated student. Scholars, faculty, fellows, and students who are visiting the UW and who are the lead researcher: identify your home institution and describe the purpose and duration of your UW visit, as well as the UW department/center with which you are affiliated while at the UW.

Note that many UW clinical faculty members are paid employees of non-UW institutions.

*The UW IRB provides IRB review and oversight for only those researchers who meet the criteria described in the **POLICY: Use of the UW IRB.***

University of Washington, Department of Psychiatry and Behavioral Sciences

1.2 Consultation history. Have you consulted with anyone at HSD about this study?

It is not necessary to obtain advance consultation. If you have: answering this question will help ensure that the IRB is aware of and considers the advice and guidance you were provided.

[ ] No
[x] Yes → If yes, briefly describe the consultation: approximate date, with whom, and method (e.g., by email, phone call, in-person meeting).

[redacted] has met with us by phone and reviewed our protocol resulting in substantial changes.

1.3 Similar and/or related studies. Are there any related IRB applications that provide context for the proposed activities?

Examples of studies for which there is likely to be a related IRB application: Using samples or data collected by another study; recruiting subjects from a registry established by a colleague's research activity; conducting Phase 2 of a multi-part project, or conducting a continuation of another study; serving as the data coordinating center for a multi-site study that includes a UW site.

Providing this information (if relevant) may significantly improve the efficiency and consistency of the IRB's review.

[ ] No
[x] Yes → If yes, briefly describe the other studies or applications and how they relate to the proposed activities. If the other applications were reviewed by the UW IRB, please also provide: the UW IRB number, the study title, and the lead researcher's name.

We have a pilot study IRB#48457 Feasibility of Pilot Survey Protocol that was designed to develop pilot online assessment protocols. The NIDA grant funding this application is a funding amendment to 48457 while we finalize those procedures. However, in proceeding with the main study, we are submitting this new proposal for the full RCT (rather than continue further as amendments to a protocol designed to be a feasibility study).

1.4 Externally-imposed urgency or time deadlines. Are there any externally-imposed deadlines or urgency that affect your proposed activity?

HSD recognizes that everyone would like their IRB applications to be reviewed as quickly as possible. To ensure fairness, it is HSD policy to review applications in the order in which they are received. However, HSD will assign a higher priority to research with externally-imposed urgency that is beyond the control of the researcher. Researchers are encouraged to communicate as soon as possible with their HSD staff contact person when there is an urgent situation (in other words, before submitting the IRB application). Examples: a researcher plans to test an experimental vaccine that has just been developed for a newly emerging epidemic; a researcher has an unexpected opportunity to collect data from students when the end of the school year is only four weeks away.

HSD may ask for documentation of the externally-imposed urgency. A higher priority should not be requested to compensate for a researcher's failure to prepare an IRB application in a timely manner. Note that IRB review requires a certain minimum amount of time; without sufficient time, the IRB may not be able to review and approve an application by a deadline.

x	**No**
	**Yes** → If yes, briefly describe the urgency or deadline as well as the reason for it.

N/A

1.5 Objectives Using lay language, describe the purpose, specific aims, or objectives that will be met by this specific project. If hypotheses are being tested, describe them. You will be asked to describe the specific procedures in a later section.

If your application involves the use of a HUD "humanitarian" device: describe whether the use is for "on-label" clinical patient care, "off-label" clinical patient care, and/or research (collecting safety and/or effectiveness data).

The goal of this study is to evaluate the effectiveness and utility of "Preventing Addiction Related Suicide" (PARS), a single session suicide prevention intervention, to increase help-seeking by clients in community addiction treatment. Evaluation is conducted through a Stepped wedge controlled trial with the following aims:

Aim 1: Compare the effectiveness of Intensive Outpatient Program (IOP) integrating PARS to TAU to change beliefs about suicide and suicide prevention.

Hypothesis 1a: Clients who receive PARS will know more accurate information about suicide

Hypothesis 1b: Clients who receive PARS will have less maladaptive attitudes about suicide

Aim 2: Compare the effectiveness of IOP integrating PARS to TAU to increase help-seeking behaviors for clients and for clients' friends or family at risk of suicide.

Hypothesis 1c: Clients who receive PARS will show greater help-seeking for themselves and others

Aim 3: Evaluate whether changes in beliefs about suicide and suicide prevention—particularly regarding warning signs for suicide, including addiction, intoxication, and relapse, as well as beliefs that suicide is preventable when action is taken—are possible mechanisms by which PARS increases help-seeking behavior.

Hypothesis 2: The effect of PARS vs. TAU on changes in help-seeking will be mediated by improved information and attitudes

Exploratory Aim 4: Evaluate possible clinic-level dose effects of PARS administration such that participant outcomes improve the longer PARS is implemented within clinics.

Exploratory Aim 5: Compare the effects of PARS vs. TAU on clients' suicidality and substance use in the follow-up period.

Administrative Supplement Aim 1: [redacted]

1.6 Study design. Provide a one-sentence description of the general study design and/or type of methodology.

Your answer will help HSD in assigning applications to reviewers and in managing workload. Examples: a longitudinal observational study; a double-blind, placebo-controlled randomized study; ethnographic interviews; web scraping from a convenience sample of blogs; medical record review; coordinating center for a multi-site study.

Pragmatic clinical trial of PARS compared to Treatment-as-Usual (TAU) using a Stepped wedge design with 900 clients enrolled in 15 community addiction treatment sites.

For those unfamiliar with Stepped wedge design, a figure is provided. The Stepped wedge design has the 15 sites randomized into 5 groups and groups are then randomly ordered from 1 to 5. At Step 1, all groups are assessed as control (i.e., TAU). Starting in Step 2, Group 1 moves from control to experimental with the 3 Group 1 sites implementing PARS and the rest continuing TAU. At each Step, the next group begins implementing PARS till by Step 6 all sites have moved to the experimental treatment. Primary outcomes compare TAU vs. PARS.

	Step 1	Step 2	Step 3	Step 4	Step 5	Step 6
Group 1 (3 sites)	TAU	PARS	PARS	PARS	PARS	PARS
Group 2 (3 sites)	TAU	TAU	PARS	PARS	PARS	PARS
Group 3 (3 sites)	TAU	TAU	TAU	PARS	PARS	PARS
Group 4 (3 sites)	TAU	TAU	TAU	TAU	PARS	PARS
Group 5 (3 sites)	TAU	TAU	TAU	TAU	TAU	PARS

Key notes regarding study setting and design: Four community substance abuse treatment agencies have partnered with the research team for this study. These four agencies have 1-9 sites each that are scattered around Western Washington for a total 15 sites. Each site varies from 1-3 Intensive Outpatient Program (IOP) groups which run simultaneously. Each IOP has approximately 24-36 sessions of 2-3 hours each (72 hours total). Once they complete all sessions, the IOP restarts. New IOP clients enter the program at any time and stay till they have attended all 24 or 36 sessions. Thus, each Step in this Stepped Wedge design will be an entirely new set of clients.

Only one IOP for each site will be selected to be the focus of this study (Selected IOP). All client participants will be recruited and consented by research staff and the interventions (TAU vs. PARS) will be in the Selected IOP.

Within each Step, data collection will be as follows. Two weeks prior to the week Standard/PARS session is to be conducted in the selected IOP, research staff will connect with counselors at that site to (a) set up a plan for client recruitment and (b) have counselors complete online assessments themselves. One week prior to the Standard/PARS session, the research staff will come to the program 1-3x to recruit participants with the goal of recruiting all willing participants enrolled in that Selected IOP. Baseline assessments completed at recruitment. Follow-up assessments online or by phone two weeks, then one, three, and six months after recruitment.

Administrative Supplement: [redacted]

1.7 Intent. Check all the descriptors that apply to your activity. You must place an "X" in at least one box.

This question is essential for ensuring that your application is correctly reviewed. Please read each option carefully.

	Descriptor
☐	1. Class project or other activity whose purpose is to provide an educational experience for the researcher (for example, to learn about the process or methods of doing research).
☐	2. Part of an institution, organization, or program's own internal operational monitoring.
☐	3. Improve the quality of service provided by a specific institution, organization, or program.
x	4. Designed to expand the knowledge base of a scientific discipline or other scholarly field of study, and produce results that:<br>• Are expected to be applicable to a larger population beyond the site of data collection or the specific subjects studied, or<br>• Are intended to be used to develop, test, or support theories, principles, and statements of relationships, or to inform policy beyond the study.
☐	5. Develop information about a drug or device through its prospective use and assignment to subjects, which will then be submitted to the Food and Drug Administration (FDA) in support of a marketing or research application for an investigational drug or device, or for changes to the purpose, population, or dose for an already-approved drug or device.
☐	6. Focus directly on the specific individuals about whom the information or biospecimens are collected through oral history, journalism, biography, or historical scholarship activities, to provide an accurate and evidence-based portrayal of the individuals.
☐	7. A quality improvement or program improvement activity conducted to improve the implementation (delivery or quality) of an accepted practice, or to collect data about the implementation of the practice for clinical, practical, or administrative purposes. This does not include the evaluation of the efficacy of different accepted practices, or a comparison of their efficacy.
☐	8. Public health surveillance activities conducted, requested, or authorized by a public health authority for the sole purpose of identifying or investigating potential public health signals or timely awareness and priority setting during a situation that threatens public health.
☐	9. Preliminary, exploratory, or research development activities (such as pilot and feasibility studies, or reliability/validation testing of a questionnaire)
☐	10. Expanded access use of a drug or device not yet approved for this purpose
☐	11. Use of a Humanitarian Use Device
☐	12. Other. Explain:

N/A

1.8 Background, experience, and preliminary work. Answer this question <u>**only**</u> if your proposed activity has one or more of the following characteristics. The purpose of this question is to provide the IRB with information that is relevant to its risk/benefit analysis.

- Involves more than minimal risk (physical or non-physical)
- Is a clinical trial, or
- Involves having the subjects use a drug, biological, botanical, nutritional supplement, or medical device.

"Minimal risk" means that the probability and magnitude of harm or discomfort anticipated in the research are not greater than those ordinarily encountered in daily life or during the performance of routine physical or psychological examinations or tests.

a. <u>Background</u>. Provide the rationale and the scientific or scholarly background for your proposed activity, based on existing literature (or clinical knowledge). Describe the gaps in current knowledge that your project is intended to address.

Do not provide scholarly citations. Limit your answer to less than one page, or refer to an attached document with background information that is no more than three pages long.

1. Suicidal behavior is prevalent and costly in substance-abusing populations.
Suicide and suicidal behaviors are over-represented in populations with substance use disorders (SUDs) compared to the general adult population. Recent reviews find that the risk of suicide is 14 times higher for people injecting drugs, 10 times for alcohol use disorders, and 17 times for polydrug users. Clients receiving alcohol treatment are about 10 times more likely to endorse of a lifetime history of suicide attempts (43%) compared to a nationally representative sample of adults (4.6%). Moreover, prospective data shows that individuals in addiction treatment had five times the odds of suicide attempt over five years compared to those not in treatment.

2. Community addiction treatment is an ideal setting for targeting suicide risk in this high-risk group.
Every year, approximately 2.5 million people in the United States enter specialized addiction treatment programs. By far, the most common modality of publicly funded addiction treatment available is group-based Intensive Outpatient Programs (IOP). Thus, adding evidence-based, transportable suicide prevention strategies into the standard IOP treatment package has the potential to reach an enormous number of people who are at very high risk for suicide. Moreover, entering addiction treatment may represent a key window for intervention to reduce suicidal behaviors, as this transition is marked by high rates of suicidal thinking and behavior. Individuals often enter addiction treatment in the context of multiple increased risk factors for suicide: when substance use is out of control and/or is resulting in particularly severe impairment (e.g., marital or financial difficulties, severe depressive symptoms). Clients with addiction also connect with each other during treatment, in twelve-step meetings, and in drug use. Improving accurate information and adaptive attitudes toward suicide prevention as well as how to effectively reduce risk and reach out for help may not only increase their access to care if suicidal, but also increase access to care of their friends and family who are often also at risk.

3. Addiction treatment providers need additional training to prevent suicidal behavior.
Unfortunately, most chemical dependency counselors feel unprepared, inadequately trained, and uncomfortable addressing the issue of suicide. This selected suicide prevention program has the potential to serve a dual purpose of providing prevention for clients and providing ongoing education and training for the addiction treatment staff tasked with delivering the program.

4. PARS was developed to be transportable, disseminable, and community-friendly.
PARS is taught as a single module that is integrated within standard IOP therapy group treatment. It was developed with suicide prevention experts as well as leaders in the addiction treatment community of Western Washington. All feasibility testing of PARS was conducted in community treatment programs.

Administrative Supplement: [redacted]

b. Experience and preliminary work. **Briefly describe experience or preliminary work or data (if any) that you or your team have that supports the feasibility and/or safety of this study.**

It is not necessary to summarize all discussion that has led to the development of the study protocol. The IRB is interested only in short summaries about experiences or preliminary work that suggest the study is feasible and that risks are reasonable relative to the benefits. Examples: You have already conducted a Phase 1 study of an experimental drug which supports the Phase 2 study you are now proposing to do; you have already done a small pilot study showing that the reading skills intervention you plan to use is feasible in an after-school program with classroom aides; you have experience with the type of surgery that is required to implant the study device; you have a study coordinator who is experienced in working with subjects who have significant cognitive impairment.

A pre-post pilot study of PARS was conducted with clients attending group-based IOP addiction treatment.

Thirteen IOP counselors were recruited and trained to administer PARS. After completing informed consent and receiving training, counselors answered a survey about PARS acceptability and utility in the standard working conditions at their sites. PARS was found to be acceptable to counselors and leadership at these agencies.

This pilot study of PARS also demonstrated significant post-intervention increases in accurate information and decreases in maladaptive attitudes toward suicide among 79 client participants. Significant gains compared to pre-intervention were maintained at 1-month follow-up for both information and maladaptive attitudes. Help-seeking was also significantly improved. Compared to the month before PARS, in the month following PARS, pilot participants were twice as likely to ask friends and family to seek help as well as to seek help themselves. This also highlights the fluidity of suicide risk during addiction treatment — although outpatient SUD treatment providers attempt to screen out acutely suicidal individuals at intake, instead referring them to a higher level of care, this is nonetheless a high-risk population and suicidal thoughts and behavior are not uncommon during community addiction treatment.

1.9 **Supplements**. Check all boxes that apply, to identify Supplements you should complete and upload to the **Supporting Documents** SmartForm in ***Zipline***.

This section is here instead of at the end of the form to reduce the risk of duplicating information in this IRB Protocol form that you will need to provide in these Supplements.

Check all That Apply	Type of Research	Supplement Name
☐	**Department of Defense** The research involves Department of Defense funding, facilities, data, or personnel.	*ZIPLINE* SUPPLEMENT: Department of Defense
☐	**Department of Energy** The research involves Department of Energy funding, facilities, data, or personnel.	*ZIPLINE* SUPPLEMENT: Department of Energy
☐	**Drug, biologic, botanical, supplement** Procedures involve the use of any drug, biologic, botanical or supplement, even if the item is not the focus of your research	*ZIPLINE* SUPPLEMENT: Drugs
☐	**Emergency exception to informed consent** Research that requires this special consent waiver for research involving more than minimal risk	*ZIPLINE* SUPPLEMENT: Exception from Informed Consent for Emergency Research (EFIC)
☐	**Genomic data sharing** Genomic data are being collected and will be deposited in an external database (such as the NIH dbGaP database) for sharing with other researchers	*ZIPLINE* SUPPLEMENT: Genomic Data Sharing
☐	**Medical device** Procedures involve the use of any medical device, even if the device is not the focus of your research, except when the device is FDA-approved and is being used through a clinical facility in the manner for which it is approved	*ZIPLINE* SUPPLEMENT: Devices
☐	**Multi-site study** (You are asking the UW IRB to review one or more sites in a multi-site study.)	*ZIPLINE* SUPPLEMENT: Participating Site in Multi-Site Research
☐	**Participant results sharing** Individual research results will be shared with subjects.	*ZIPLINE* SUPPLEMENT: Participant Results Sharing
X	None of the above	

2 PARTICIPANTS

2.1 **Participants**. Describe the general characteristics of the subject populations or groups, including age range, gender, health status, and any other relevant characteristics.

Client participants (N=900) will be adults with a substance use disorder (SUD) that resulted in their admission to the selected IOP (Selected IOP). All clients enrolled in the Selected IOP will be recruited to participate.

Counselor participants (N=~50, maximum 200) are chemical dependency providers and/or clinicians working at the study sites. To determine effects of PARS implementation beyond the Selected IOP all counselors who work at all the sites will be recruited to participate including any counselors newly hired across the course of the study.

Administrative Supplement: [redacted]

2.2 Inclusion and exclusion criteria. Describe the specific criteria you will use to decide who will be included in your study from among interested or potential subjects. Define any technical terms in lay language.

Assumed by virtue of placement that they would be eligible (refer to section 4.1)

Client Participant Inclusion Criteria

1. Enrolled client in one of the community treatment settings
2. Over 18 years of age (no maximum age)
3. Ability to understand written and spoken English

Client Participant Exclusion Criteria

1. Any clinical medical/psychiatric condition, severity of that condition, or life situation that in the opinion of the counselors or Drs. Comtois or Ries would compromise safe and voluntary study participation (e.g., psychosis, custody conflict). This is expected to be a rare circumstance and will be known prior to the recruitment session. If a counselor does not want someone involved, they will not be. If counselor is unsure, Dr. Comtois or Ries will facilitate decision with counselor ahead of time to assist in the decision.

Counselor Participants – Inclusion Criteria:

1. Hired as regular staff counselor at one of the partner agencies
2. "Engagement staff": anyone who has a clinical treatment relationship with clients (CDPs, counselors, etc.).
3. Ability to understand written and spoken English

Counselor Participants – Exclusion Criteria:

1. Determined by agency administrator as not appropriate to participate (do not have specific expectation this will occur but want to provide agencies with possibility of excluding counselor at their discretion)

Stakeholder Participants – Inclusion Criteria:

1. Stakeholders for the Administrative Supplement will be [redacted]

Stakeholder Participants – Exclusion Criteria:

1. None

2.3 Prisoners. IRB approval is required in order to include prisoners in research, even when prisoners are not an intended target population.

a. Will you recruit or obtain data from individuals that you know to be prisoners?

For records reviews: if the records do not indicate prisoner status and prisoners are not a target population, select "No". See the WORKSHEET: Prisoners for the definition of "prisoner".

- [x] No
- [ ] Yes → If yes, answer the following questions (i – iv).

i. Describe the type of prisoners, and which prisons/jails:

N/A

ii. One concern about prisoner research is whether the effect of participation on prisoners' general living conditions, medical care, quality of food, amenities, and opportunity for earnings in prison will be so great that it will make it difficult for prisoners to adequately consider the research risks. What will you do to reduce the chances of this?

N/A

iii. Describe what you will do to make sure that (a) your recruitment and subject selection procedures will be fair to all eligible prisoners and (b) prison authorities or other prisoners will not be able to arbitrarily prevent or require particular prisoners from participating.

N/A

iv. If your research will involve prisoners in federal facilities or in state/local facilities outside of Washington State: check the box below to provide your assurance that you will (a) not encourage or facilitate the use of a prisoner's participation in the research to influence parole decisions, and (b) clearly inform each prisoner in advance (for example, in a consent form) that participation in the research will have no effect on his or her parole.

- [ ] Confirmed

b. Is your research likely to have subjects who become prisoners while participating in your study?

For example, a longitudinal study of youth with drug problems is likely to have subjects who will be prisoners at some point during the study.

- [ ] No
- [x] Yes → If yes, if a subject becomes a prisoner while participating in your study, will you continue the study procedures and/or data collection while the subject is a prisoner?
  - [ ] No
  - [x] Yes → If yes, describe the procedures and/or data collection you will continue with prisoner subjects

If we know someone is a prisoner, we will not continue follow-up assessments until after their release. However, it is possible (although unlikely) that our text message or email and online follow-up assessment system will reach a participant while a prisoner (e.g. in King County jail) and we do not know that they are. This is unlikely as cell phone and computer access is not available during incarceration. Most likely these messages will be discovered by the participant after release at which time they are no longer

prisoners. While there is no easy way to prevent this occurrence, we do not believe there is any way the participant would feel coerced into participation so the risk to the participant would be low and there is no way that this could have a role in parole or other legal decisions.

In order to ensure this, our consent form also contains this information. The client consent form states that "If you become incarcerated over the course of the study, we will not be able to contact you or collect any information from you until you are released. Depending on the circumstances, it may be possible that we will need to stop any further follow up assessments".

2.4 Protected populations. IRB approval is required for the use of the subject populations listed here. Check the boxes for any of these populations that you will purposefully include in your research. (In other words, being a part of the population is an inclusion criterion for your study.)

The WORKSHEETS describe the criteria for approval but do not need to be completed or submitted.

Population	Worksheet
☐ Children	WORKSHEET: Children
☐ Children who are wards	WORKSHEET: Children
☐ Fetuses in utero	WORKSHEET: Pregnant Women
☐ Neonates of uncertain viability	WORKSHEET: Neonates
☐ Non-viable neonates	WORKSHEET: Neonates
☐ Pregnant women	WORKSHEET: Pregnant Women

"Children" are defined as individuals who have not attained the legal age for consent to treatments or procedures involved in the research and its specific setting. This will vary according to the location of the research (that is, for different states and countries).

a. If you check any of the boxes above, use this space to provide any information you think may be relevant for the IRB to consider.

N/A

2.5 Native Americans or non U.S. indigenous populations. Will you actively recruit from Native American or non-U.S. indigenous populations through a tribe, tribe-focused organization, or similar community-based organization?

Indigenous people are defined in international or national legislation as having a set of specific rights based on their historical ties to a particular territory and their cultural or historical distinctiveness from other populations that are often politically dominant.

Examples: a reservation school or health clinic; recruiting during a tribal community gathering

☐ No

X	Yes	→ If yes, name the tribe, tribal-focused organization, or similar community based organization. The UW IRB expects that you will obtain tribal/indigenous approval before beginning your research.

[redacted] (representing a large number of Alaska Native and American Indian tribes in the [redacted]

2.6 Third party subjects. Will you collect private identifiable information about *other individuals* from your subjects? Common examples include: collecting medical history information or contact information about family members, friends, co-workers.

"Identifiable" means any direct or indirect identifier that, alone or in combination, would allow you or another member of your research team to readily identify the person. For example, suppose that you are studying immigration history. If you ask your subjects several questions about their grandparents but you do not obtain names or other information that would allow you to readily identify the grandparents, then you are not collecting private identifiable information about the grandparents.

X	No	
	Yes	→ If yes, these individuals are considered human subjects in your study. Describe them and what data you will collect about them.

2.7 Number of subjects. Can you predict or describe the maximum number of subjects (or subject units) you need to complete your study, for each subject group?

Subject units mean units within a group. For most research studies, a group will consist of individuals. However, the unit of interest in some research is not the individual. Examples:

- *Dyads such as caregiver-and-Alzheimer's patient, or parent and child*
- *Families*
- *Other units, such as student-parent-teacher*

Subject group means categories of subjects that are meaningful for your research. Some research has only one subject group – for example, all UW students taking Introductory Psychology. Some common ways in which subjects are grouped include:

- *By intervention – for example, an intervention group and a control group.*
- *By subject population or setting – for example, urban versus rural families*
- *By age – for example, children who are 6, 10, or 14 years old.*

The IRB reviews the number of subjects you plan to study in the context of risks and benefits. You may submit a Modification to increase this number at any time after you receive IRB approval. If the IRB determines that your research involves no more than minimal risk: you may exceed the approved number and it will not be considered non-compliance. If your research involves more than minimal risk: exceeding the approved number will be considered non-compliance.

	No	→ If no, provide your rationale in the box below. Also, provide any information you can about the scope/size of the research. You do not need to complete the table.

Example: you may not be able to predict the number of subjects who will complete an online survey advertised through Craigslist, but you can state that you will post your survey for two weeks and the number who respond is the number who will be in your study.

N/A

x **Yes** → If yes, for each subject group, use the table below to provide your estimate of the maximum desired number of individuals (or other subject unit, such as families) who will complete the research.

Group name/description	Maximum desired number of individuals (or other subject unit, such as families) who will complete the research<br>**For clinical trials: provide numbers for your site and for the study-wide total number*
Treatment as Usual (TAU)	450
PARS (experimental condition)	450
Counselor Participants	200
Stakeholder Participants for Administrative Supplement	

3 INTERNATIONAL RESEARCH SETTING

Answer the questions in this section ONLY if your research will occur at sites outside of the United States

3.1 Reason for sites. Describe the reason(s) why you selected the sites where you will conduct the research.

N/A

3.2 Local context. Culturally-appropriate procedures and an understanding of local context are an important part of protecting subjects. Describe any site-specific cultural issues, customs, beliefs, or values that may affect your research or how it is conducted.

Examples: It would be culturally inappropriate in some international settings for a woman to be directly contacted by a male researcher; instead, the researcher may need to ask a male family member for permission before the woman can be approached. It may be appropriate to obtain permission from community leaders prior to obtaining consent from individual members of a group.

This federal site maintains an international list of human research standards and requirements: http://www.hhs.gov/ohrp/international/index.html

N/A

3.3 Site-specific laws. Describe any local laws that may affect your research (especially the research design and consent procedures). The most common examples are laws about:

- **Specimens** – for example, some countries will not allow biospecimens to be taken out of the country.
- **Age of consent** – laws about when an individual is considered old enough to be able to provide consent vary across states, and across countries.
- **Legally authorized representative** – laws about who can serve as a legally authorized representative (and who has priority when more than one person is available) vary across states and countries.
- **Use of healthcare records** – many states (including Washington State) have laws that are similar to the federal HIPAA law but that have additional requirements.

N/A

3.4 Site-specific administrative or ethical requirements. Describe local administrative or ethical requirements that affect your research.

Example: A school district may require you to obtain permission from the head district office as well as school principals before approaching teachers or students; a factory in China may allow you to interview factory workers but not allow you to pay them.

N/A

4 RECRUITING and SCREENING PARTICIPANTS

4.1 Recruiting and Screening. Describe how you will identify, recruit, and screen subjects. Include information about: how, when, where, and in what setting. Identify who (by position or role, not name) will approach and recruit subjects, and who will screen them for eligibility.

Client Participant Recruitment:
A week ahead, counselors will inform clients from each selected IOP that research staff will be coming by providing them the PARS client recruitment flyer (attached). Using the same flyer a week later, all clients will be reminded about the study and told to stay at the end of a group if they would like to hear more about it.

Using the recruitment flyer for talking points, the research staff will come at the end of group to explain what the study is and recruit participants.

(If a client participant does not meet study criteria, the IOP counselor will call that client aside as the IOP group ends to explain this to them privately and the client will not stay to hear about the study or participate.)

Counselor Participant Recruitment:
The agency leadership will inform counselors from each site that research staff will be coming by providing them the PARS counselor recruitment flyer (attached).

Using the recruitment flyer for talking points, the research staff will come to explain what the study is and recruit counselor participants.

All counselors (defined as staff with a treatment relationship with clients) will be invited to participate at the beginning of the study. At each subsequent Step, if new counselors have started they will be recruited at that time and will complete the remainder of the study.

(If a counselor participant does not meet study criteria, the agency staff will call that counselor aside to explain this to them privately and the counselor will not stay to hear about the study or participate.)

Stakeholder Recruitment for the Administrative Supplement: [redacted].

4.2 Recruitment materials.

a. What materials (if any) will you use to recruit and screen subjects?

Examples: talking points for phone or in-person conversations; video or audio presentations; websites; social media messages; written materials such as letters, flyers for posting, brochures, or printed advertisements; questionnaires filled out by potential subjects.

As noted in 4.1 above, there will be two recruitment flyers – one for client participants and one for counselor participants. Both are attached.

We would like to request flexible approval of recruitment strategy and materials, specifically to create or update recruitment materials without submitting a modification to the application so long as the overall content does not go outside the scope and range of what is already approved. Any substantive change to content would be submitted as a formal modification.

b. Upload descriptions of each type of material (or the materials themselves) to the **Consent Forms and Recruitment Materials** SmartForm of ***Zipline***. If you will send letters to the subjects, the letter should include a statement about how you obtained the subject's name, contact information, and any other subject-specific information (such as a health condition) that is mentioned in the letter.

HSD encourages researchers to consider uploading descriptions of most recruitment and screening materials instead of the materials themselves. The goal is to provide the researchers with the flexibility to change some information on the materials without submitting a Modification for IRB approval of the changes. Examples:

- *You could provide a list of talking points that will be used for phone or in-person conversations instead of a script.*
- *For the description of a flyer, you might include the information that it will provide the study phone number and the name of a study contact person (without providing the actual phone number or name). In doing so, you would not need to submit a Modification if/when the study phone number or contact person changes. Also, instead of listing the inclusion/exclusion criteria, you might state that the flyer will list one or a few of the major inclusion/exclusion criteria.*
- *For the description of a video or a website, you might include a description of the possible visual elements and a list of the content (e.g., study phone number; study contact person; top three inclusion/exclusion criteria; payment of $50; study name; UW researcher).*

4.3 Relationship with participant population. Do any members of the study team have an existing relationship with the study population(s)?

Examples: a study team member may have a dual role with the study population (for example, being their clinical care provider, teacher, laboratory directory or tribal leader in addition to recruiting them for his/her research).

	No
X	Yes → If yes, describe the nature of the relationship.

Dr. Ries works extensively with substance abuse treatment sites in Western Washington and has or may consult with them on challenging cases etc. over the next five years. It is unlikely that one of the client participants will be someone he consults on, but this is not impossible. It is somewhat more likely that he would consult with a counselor participant. These consultations are at the agency's request and they are under no obligation to do so.

Dr. Ries also has a longstanding consultation relationship with the [redacted] Indian Tribe. Dr. Comtois has been conducting another research study with both the [redacted] in which we have conducted a cultural tailoring process comparable to the one proposed here.

4.4 Payment to participants. Describe any payment you will provide, including:

- The total amount/value
- Whether payment will be "pro-rated" so that participants who are unable to complete the research may still receive some part of the payment

The IRB expects the consent process or study information provided to the subjects to include information about the number and amount of payments, and especially the time when subjects can expect to receive payment. One of the most frequent complaints received by HSD is from subjects who expected to receive cash or a check on the day that they completed a study and who were angry or disappointed when payment took 6-8 weeks to reach them.

Do not include a description of any expenses that will be reimbursed.

Client participants will be reimbursed $30 (in a choice of gift cards) for the baseline assessment. The initial client follow-up assessment one week after the Standard/PARS session is shorter so reimbursed at $20. The 1, 3 and 6 month follow-up assessments are reimbursed $30 each.

In addition, to minimize attrition, client participants will also be offered an additional incentive to be paid at the final 6-month assessment (or end of the 6-month assessment window if they do not complete it). The additional incentive (also in gift cards) will be $20 for completing 2 of the 3 outcome assessments and $30 for completing all outcome assessments.

Counselor participants will be reimbursed $30 (in a choice of gift cards) for the baseline and follow-up assessments (i.e. every four months). Additionally, the counselor leading the study PARS group will receive an additional $20 for completing the brief acceptability questionnaire after the first PARS group.

Stakeholder Participants [redacted]

4.5 Non-monetary compensation. Describe any non-monetary compensation you will provide. Example: extra credit for students; a toy for a child. If you will be offering class credit to students, you must provide (and describe) an alternate way for the students to earn the extra credit without participating in your research.

As reimbursement for attending and completing PARS Training, counselor participants will receive free National Association of Social Work CE Credits for the hours they participate in the PARS training.

4.6 Consent for recruiting and screening. Will you obtain consent for any of the recruiting and screening procedures? (Section 8: Consent of Adults asks about consent for the main study procedures).

"Consent" includes: consent from individuals for their own participation; parental permission; assent from children; consent from a legally authorized representative for adult individuals who are unable to provide consent.

Examples:

- *For a study in which names and contact information will be obtained from a registry: the registry should have consent from the registry participants to release their names and contact information to researchers.*
- *For a study in which possible subjects are identified by screening records: there will be no consent process.*
- *For a study in which individuals respond to an announcement and call into a study phone line: the study team person talking to the individual may obtain non-written consent to ask eligibility questions over the phone.*

[ ]	No	→ If no, you must still answer question 4.7 below.
X	Yes	→ If yes, describe the consent process.

There is no eligibility screening. See Section 2.1 and 4.1.

a. Documentation of consent. Will you obtain a written or verifiable electronic signature from the subject on a consent form to document consent for all of the **recruiting and screening procedures**?

[ ]	No	→ If no, describe the information you will provide during the consent process and for which procedures.
[ ]	Yes	→ If yes, upload the consent form to the **Consent Forms and Recruitment Materials** page of ***Zipline***.

4.7 Data and specimens for recruiting and screening. For studies where you will obtain consent, describe any data and/or specimens (including any PHI) you will obtain for recruiting and screening (prior to obtaining consent) and whether you will retain it as part of the study data.

Obtain *means to possess or record in any fashion (writing, electronic document, video, email, voice recording, etc.) for research purposes and to retain for any length of time.*

Examples: names and contact information; the information gathered from records that were screened; results of screening questionnaires or screening blood tests; Protected Health Information (PHI) from screening medical records to identify possible subjects.

There is no eligibility screening. See Section 2.1 and 4.1.

5 PROCEDURES

5.1 Study procedures. Using lay language, provide a complete description of the study procedures, including the sequence, intervention or manipulation (if any), time required, and setting/location. If it is available and you think it would be helpful to the IRB: Upload a study flow sheet or table to the **Supporting Documents** SmartForm in ***Zipline***.

*For studies comparing standards of care: It is important to accurately identify the research procedures. See UW IRB **POLICY: Risks of Harm from Standard Care** and the draft guidance from the federal Office of Human Research Protections, **"Guidance on Disclosing Reasonably Foreseeable Risks in Research Evaluating Standards of Care";** October 20, 2014.*

Study Interventions

In this study, we are using a Stepped wedge design that randomizes at the site level. That means that all clients will receive whichever intervention their site is providing at the time they enroll. This will be Treatment as Usual (TAU) until the Stepped wedge design randomization selects that site to implement PARS at which point all clients will receive PARS till the end of the study. This will be true for all clients and counselors whether or not they choose to participate in the study. Thus, the study is restricting the choice of interventions for study participants and non-participants.

This is described in the consent form for participants. However, because of the study design, non-subjects will also experience randomization and exposure to the intervention. We request a waiver of consent for this.

Clarification of IOP Treatment in which both study interventions are embedded

Intensive Outpatient Program (IOP) guidelines in Washington State are based on State requirements. Within these guidelines, agencies typically offer IOP consisting of three-hour groups, three times a week, over eight weeks, for a total of 24 groups (72 hours total). Some programs meet less often for a longer time window up to 12 weeks. The 24-36 session series runs and then starts over continuously. Clients can enter the IOP at any time in the 24 session series and stay until they have attended all 24-36 sessions. Thus, within 24-36 sessions, all IOP clients will have completed (or dropped) from the program and a new group of IOP participants will be in the program. In our Stepped wedge design, each step is 4 months, which means that when we return to the site at each step, we will be recruiting from a different pool of clients.

Although at least one monthly individual counseling session is required, the primary modality is a group format. Each group session is 2-3 hours long and a combination of didactic information and process discussion. IOP programs are required to provide education on specific topics, e.g., alcohol and drug education, relapse prevention, risks of drug or alcohol use during pregnancy, blood borne pathogens (including HIV/AIDS and Hepatitis), emotional, physical, and sexual abuse, and nicotine addiction. However, within a 24-36-session curriculum, IOP programs have wide latitude in determining group content since the required topics typically represent less than 50% of the 24-36 sessions. Therefore the content in IOP programs across our sites is expected to be variable while the structure of session hours will be consistent. While IOP programs from our partner agencies include mental health topics, currently no IOP includes a suicide prevention focused session.

Control: Treatment as Usual (TAU)

At the beginning of the study, each site will select a particular group session in their 24-36-session schedule to be their TAU session for this study. To minimize variability between sites, the TAU session will be one regarding grief, depression, or coping with negative emotions. All sites have a TAU session on

one of these topics. They will continue to present this TAU session at the same point in the 24-36-session schedule as long as their site is randomized to TAU.

Intervention: Preventing Addiction Related Suicide (PARS)

At the Step where the site is randomized, that site will begin implementing Preventing Addiction Related Suicide (PARS) instead of the identified TAU session.

PARS is a single session that includes a specified combination of didactic presentations and group discussions. PARS topics include: Goals and Objectives; Suicide Overview; Addiction and Suicide: A Strong Relationship; Suicide Myths and Facts; Suicide Risk Factors; Suicide Protective Factors; Common Triggers of Suicidal Thoughts and Behaviors; Warning Signs and Guidelines for Preventing Addiction Related Suicide. The PowerPoint slides of the PARS curriculum and the PARS counselor adherence measure are attached.

All IOP counselors will be trained in the Step where they are randomized to implement PARS. In this way, the study sites are "engaged" in the research as counselors on site will provide the study interventions. The agencies will be deferring human subjects review to the UW and have completed an Institutional Agreement for IRB Review (IAIR) with each site.

Dr. Ries, who developed PARS, will provide all the training. To maximize the value to our community partners and assure a counselor trained in PARS is available on the day the PARS module is scheduled, all consenting counselors will be offered the opportunity to participate in the training so that someone can substitute if the designated PARS counselor is unavailable.

PARS counselor adherence will be evaluated at the first PARS administration by having an adherence coder sit in on the PARS session. Observation is the method requested by the agency treatment partners as least disruptive and most acceptable to clients. As adherence coders will have participant contact and be hearing the treatment session, they will complete any training on specific agency procedures regarding treatment observers with regard to client PHI so the adherence coders meet all agency standards. No client PHI will be collected as part of adherence coding – the focus is on the counselor's level of adherence to PARS and not client identity nor comments.

Both Conditions:

Any additional treatments outside of the IOP program (e.g., mental health-oriented counseling, pharmacotherapy) will be available during all phases of the Stepped design. Washington State mandates self-help group attendance in addition to attendance of group and individual IOP sessions; this will also remain consistent throughout each site's participation (regardless of when PARS is implemented at each site).

Client Participant Assessment Procedures:

Reminder – clinics are randomized at the clinic level. Clients and counselors have no choice in if they receive the intervention. This randomization effects clients and counselors regardless of whether they are study participants or not. As noted above, participants are informed about this in the consent form and we request waiver of consent for the non-participants.

Baseline Assessment

Will always occur one week prior to session intervention.

Will be conducted by in-person group administration. Assessments will be completed on paper or tablet computers. Expected to take 15-30 minutes.

If the individual does not have time or requests an individual assessment, this will be offered at a later time or in a different room at the same time.

Post-Intervention Assessment

Will be conducted one week after the PARS or TAU group session (i.e., 2 weeks after baseline). Assessments will be completed using online assessment. Participant will receive a text message or email (their preference) with a link to the assessment. If a participant prefers, follow-up assessments can be conducted by phone in which case research staff will call participant when the assessment is due. Expected to take 5-10 minutes.

Long-Term Follow-Up Assessments

Will be conducted 1, 3, and 6 months after the baseline assessment. Participants will receive a text message or email (their preference) with a link to the assessment. If a participant prefers, follow-up assessments can be conducted by phone in which case research staff will call participant when the assessment is due. Expected to take 10-15 minutes.

Following their completion of the 6-month assessment, participants have the option of checking a box—or verbally agreeing if the survey is conducted via phone—at the end of the survey to request the correct answers to the PARS Suicide Knowledge Scale questions (description of measure in Section 5.13 of current protocol). We will not provide the participant's individual survey responses during this process.

Counselor Participant Assessment Procedures:

Baseline Assessment

Will be conducted by in-person group administration. Assessments will be completed on tablet computers or paper versions. Expected to take 10-20 minutes.

If the individual does not have time or requests an individual assessment, this will be offered at a later time or in a different room at the same time.

Additionally, at the beginning of each Step, the Research Coordinator (or site-assigned RA) will reach out to the Site Administrator and Treatment Coordinator to ask if any new clinical staff have joined (or left) the site since the last Step. To clarify who was there previously, the Research Coordinator will provide a list of counselors who have been approached (regardless of whether they completed or refused).

Post-Training and Post-Intervention Follow-Up Assessment

Will be conducted immediately after the PARS training (when this is scheduled according to the Stepped wedge design) and immediately after the first administration of PARS, only for the counselor leading the PARS session. Assessments will be completed using online assessment using a tablet computer or paper version. Expected to take 5-10 minutes.

Ongoing Follow-Up Assessments

Will be conducted every 4 months (i.e., at each Step in the Stepped wedge) and one at the end of the study (after Step 6). Participants will receive a text message or email (their preference) with a link to the assessment. If a participant prefers, follow-up assessments can be conducted by phone in which case research staff will call participant when the assessment is due. Expected to take 10-15 minutes. (NOTE: for Step 1, counselors consented to 6 assessments and they were every 6 months. They also did not consent to the assessment at the end of the study. The PARS counselor Step 1 Re-Consent IRB ver-1 8-

21-17 document clarifies the questions to be asked at the Step 2 follow-up to re-consent these counselor participants. Also noted in Section 8.)

Study Locations

Location	Activities
Harborview Pat Steel Building	Study management, data management, entry, and analysis
Evergreen Recovery	Main Agency
Everett Outpatient	Recruitment, baseline assessment, study intervention
Lynnwood Outpatient	Recruitment, baseline assessment, study intervention
Lakeside Milam Recovery Center	Main Agency
Auburn	Recruitment, baseline assessment, study intervention
Edmonds	Recruitment, baseline assessment, study intervention
Everett	Recruitment, baseline assessment, study intervention
Issaquah	Recruitment, baseline assessment, study intervention
Kirkland	Recruitment, baseline assessment, study intervention
Puyallup	Recruitment, baseline assessment, study intervention
Renton	Recruitment, baseline assessment, study intervention
Seattle	Recruitment, baseline assessment, study intervention
Tacoma	Recruitment, baseline assessment, study intervention
Northwest Integrated Health	Main Agency
Tacoma	Recruitment, baseline assessment, study intervention
Puyallup	Recruitment, baseline assessment, study intervention
Lakewood	Recruitment, baseline assessment, study intervention
Olalla Recovery Centers	Main Agency
Gig Harbor	Recruitment, baseline assessment, study intervention

Note that for the Administrative Supplement, we are describing the PARS intervention to them but there is no trial of the intervention, *per se*.

5.2 **Data variables.** Describe the specific data you will obtain (including a description of the most sensitive items). If you would prefer, you may upload a list of the data variables to the **Supporting Documents** SmartForm instead of describing the variables below.

We will collect questionnaire data on REDCap on a tablet or paper (baseline) or online (follow-up). Follow-up interviews will be conducted by phone if so requested by a participant. The content of the questionnaires and description of most sensitive items are described below in Section 5.13.

Our goal is to attempt recruitment of all counselors at each site at each Step of the study. At Steps 2-6, the study therefore reaches out to the site's point of contact to determine if there are any counselors who have left or new counselors hired since the previous Step. New counselors can then be recruited. To prevent inadvertent re-recruitment of counselors who refused to participate at an earlier Step: we request to retain the names of counselors who refuse so that the research staff can provide a complete list of counselors from the previous Step to the site point of contact and clarify who refused so they won't be re-recruited.

We will also obtain the medical record for each participant for their treatment at the site that includes the IOP program and an ancillary services. This will allow us to determine how many IOP sessions they attended and whether they completed the program, what other services they received, what diagnoses and problems were identified for that client and whether they resolved during the course of treatment, and what their disposition plan was following the IOP program. If any of this information is not clear in the medical record, a research assistant will ask the counselor to clarify or add needed information verbally.

We believe that losing a client to suicide at a site could change the responses that participants give at a given Step of the study. Therefore, at the beginning of each step, we will ask if a client at the site died by suicide since our last assessment and if so, was it someone in the target group from which we are recruiting client participants. (We may well learn from other sources that our participant died by suicide (i.e., when they do not respond to outcome assessments), but the purpose of this procedure is to be aware of a potential confounder of study results – that is, that a suicide at the site might change suicide-related study results at that Step. Of course, if we learn a study participant then we will submit an adverse event report to the IRB.)

For the Administrative Supplement, [redacted]

5.3 Data sources. For all types of data that you will access or collect for this research: Identify whether you are obtaining the data from the subjects (or subjects' specimens) or whether you are obtaining the data from some other source (and identify the source).

If you have already provided this information in Question 5.1, you do not need to repeat the information here.

Participant questionnaires, medical record (plus verbal consultation with counselor, if needed)

For the Administrative Supplement, [redacted]

5.4 Retrospective/prospective. For all types of data and specimens that you will access or collect for this research: Describe which data are:

- Retrospective (i.e., exist at the time when you submit this application)
- Prospective (i.e., do not yet exist at the time when you submit this application)
- Both retrospective and prospective (for example, past and future school records)

Retrospective. (NOTE: Their medical history in the records preceeds study constent; but their records are obtained after consent.)

5.5 Identifiability of data and specimens. Answer these questions carefully and completely. This will allow HSD to accurately determine the type of review that is required and to assist you in identifying relevant compliance requirements. Review the following definitions before answering the questions:

Access *means to view or perceive data, but not to possess or record it. See, in contrast, the definition of "obtain".*
Identifiable *means that the identify of an individual is or may be readily (1) ascertained by the researcher or any other member of the study team from specific data variables or from a combination of data variables, or (2) associated with the information.*
Direct identifiers *are direct links between a subject and data/specimens. Examples include (but are not limited to): name, date of birth, medical record number, email or IP address, pathology or surgery accession number, student number, or a collection of your data that is (when taken together) identifiable.*
Indirect identifiers *are information that links between direct identifiers and data/specimens. Examples: a subject code or pseudonym.*
Key *refers to a single place where direct identifiers and indirect identifiers are linked together so that, for example, coded data can be identified as relating to a specific person. Example: a master list that contains the data code and the identifiers linked to the codes.*
Obtain *means to possess or record in any fashion (writing, electronic document, video, email, voice recording, etc.) for research purposes and to retain for any length of time. This is different from* ***accessing****, which means to view or perceive data.*

5.10 Communication with subjects during the study. Describe the types of communication (if any) you will have with already-enrolled subjects during the study. Provide a description instead of the actual materials themselves.

Examples: email, texts, phone, or letter reminders about appointments or about returning study materials such as a questionnaire; requests to confirm contact information.

Participants will be contacted by text message (using Twillio within the REDCap system) or email (their preference) with a link to the online REDCap survey unless they request contact by phone to do a phone interview. If they do not complete the survey when expected, research staff will contact them to remind them, solve any technical difficulties, or complete the assessment by phone. Using the follow-up locating consent form and associated contact information form (attached), research staff will reach out through the means they approve to locate them for follow-up interviews. This includes phone, email, texts, letters, private messages to social media, or contacting a friend or treatment provider they authorized us to speak to. (As noted in 8.2b below, we separate the follow-up locating consent from the main consent so that we can show it to people providing contact information to assure them we have the participant's permission without sharing any details about the study.)

5.11 Future contact with subjects. Do you plan to retain any contact information you obtain for your subjects so that they can be contacted in the future?

	No
X	Yes

→ If yes, describe the purpose of the future contact, and whether use of the contact information will be limited to your team; if not, describe who else could be provided with the contact information. Describe your criteria for approving requests for the information.

Examples: inform subjects about other studies; ask subjects for additional information or medical record access that is not currently part of the study proposed in this application; obtain another sample.

Client participants opted in/out of the following statement at the time of consent: "It is possible that, depending on the results of the study, the research team may want to interview you again in the future. Please initial here ______ if you are willing to be contacted in the future about participation in a follow-up study. You are not required to participate in further research if contacted in the future." No such study, nor contact with participants, will be performed without explicit review and approval by UW HSD. Only the research team will have this information. It will not be shared.

5.12 Alternatives to participation. Are there any alternative procedures or treatments that might be advantageous to the subjects?

If there are no alternative procedures or treatments, select "No". Examples of advantageous alternatives: earning extra class credit in some time-equivalent way other than research participation; obtaining supportive care or a standard clinical treatment from a health care provider instead of participating in research with an experimental drug.

X	No
	Yes

→ If yes, describe the alternatives.

N/A

5.13 Upload to the Supporting Documents SmartForm of ***Zipline*** **all data collection forms (if any) that will be directly used by or with the subjects, and any scripts/talking points you will use to collect the data. Do not include data collection forms that will be used to abstract data from other sources (such as medical or academic records, or video recordings.**

- ***Examples***: *survey, questionnaires, subject logs or diaries, focus group questions.*
- ***NOTE:*** *Sometimes the IRB can approve the general content of surveys and other data collection instruments rather than the specific form itself. This prevents the need to submit a modification request for future minor changes that do not add new topics or increase the sensitivity of the questions. To request this general approval, use the text box below to identify the questionnaires/surveys/ etc. for which you are seeking this more general approval. Then briefly describe the scope of the topics you will cover and the most personal and sensitive questions. The HSD staff person who screens this application will let you know whether this is sufficient or whether you will need to provide more information.*
- ***For materials that cannot be uploaded***: *upload screenshots or written descriptions that are sufficient to enable the IRB to understand the types of data that will be collected and the nature of the experience for the participant. You may also provide URLs (website addresses) or written descriptions below. Examples of materials that usually cannot be uploaded: mobile apps; computer-administered test; licensed and restricted standardized tests.*
- ***For data that will be gathered in an evolving way***: *This refers to data collection/questions that are not pre-determined but rather are shaped during interactions with participants in response to observations and responses made during those interactions. If this applies to your research, provide a description of the process by which you will establish the data collection/questions as you interact with subjects, how you will document your data collection/questions, the topics you plan to address, the most sensitive type of information you will plan to gather, and the limitations (if any) on topics you will raise or pursue.*

Use this text box (if desired) to provide:

- Short written descriptions of materials that cannot be uploaded, such as URLs
- A description of the process you will use for data that will be gathered in an evolving way.
- The general content of questionnaires, surveys and similar instruments for which you are seeking general approval. (See the **NOTE** bullet point in the instructions above.)

We would like to request flexible approval of recruitment instruments. Specifically, we would like to request that the IRB will approve the general content of our questionnaires rather than the specific forms themselves. This will allow us to create or update recruitment instruments without submitting a modification to the application - so long as the overall content does not go outside the scope and range of what is already approved. Any substantive change to content would be submitted as a formal modification.

Please find below a description of each questionnaire and the most sensitive item for each.

Client and Counselor Measures:

Accurate Information about suicide. The PARS Suicide Knowledge Scale assesses factual understanding of warning signs, triggers, and interventions for suicide. Items include: *"Relapsing on drugs or alcohol is a common trigger for suicidal thoughts/feelings. 1 = True , 2 = False, 99 = I cannot/do not want to answer"*

Attitudes toward suicide. The PARS Attitude Scale, originally adapted from the 14-item Staff Suicide Prevention Survey, is rated on a Likert-type scale from 1 (Strongly Disagree) to 5 (Strongly Agree). This scale evaluates stigma and bias toward suicidal acts or persons, as well as perceptions that suicide is preventable if appropriate action is taken. Items include: *"Talking about suicide might give a person unwanted ideas about suicide."*

PARS Demographic Form: The PARS Demographic Form assesses demographic information to better classify clients and counselor identifying criteria (Age, Gender, etc.). Most sensitive item on client version: *"In the past 5 years, have you had consensual sex with". Is rated on the criteria of: Only females…Only males… Both*

males and females… No consensual sex past 5 years… I cannot/do not want to answer." Most sensitive version on counselor version: *"Do you consider yourself to be: Heterosexual/straight… Gay/lesbian/homosexual… Bisexual… Other Describe: __________ …I cannot/do not want to answer"*

Client Only Measures:

Help-seeking behavior. The PARS Behavior Scale – Client Version consists of four items assessing help-seeking behavior for self and others. Participants report the frequency of help-seeking behavior from never ("0 times/none") to "more than 3 times." Items include: *"In the past month, have you: (1)… asked a friend to get help because you were worried that he or she was having suicidal thoughts/feelings; (2)… asked a family member or relative to get help because you were worried he or she was having suicidal thoughts/feelings; (3)… asked for help because you were having suicidal thoughts/feelings; and (4)… called a crisis line/suicide hotline?"* If items 1 to 3 are endorsed, participants will also be asked to report whom they asked for help.

Suicidal ideation and behavior. At baseline, we will as about a lifetime history of suicidal and non-suicidal self-harm. Items will include *"Have you ever thought about or attempted to kill yourself?. With responses of: Never…It was just a brief passing thought…I have had a plan at least once to kill myself but did not try to do it …I have had a plan at least once to kill myself and really wanted to die…I have attempted to kill myself, but did not want to die…I have attempted to kill myself, and really hoped to die…I cannot/do not want to answer".* At each follow-up, we will ask similar question, but only pertaining to a history of suicidal and non-suicidal behaviors since the last assessment. Items will include *"How often have you thought about killing yourself IN THE PAST MONTH? : 0=Never, 1=Rarely (1 time), Sometimes (2 times) 3=Often (3-4 times), 4=Very Often (5 or more times)".*

Drug and alcohol use. To assess days of drug/alcohol use in past 30 days will ask clients to self-report a numerical value of "*How many days in the past 30 have you used alcohol? _____*" and "*How many days in the past 30 have you used drugs? _____*".

To assess baseline substance use problems, participants will complete a modified version of the Alcohol, Smoking and Substance Involvement Screening Test (ASSIST), a modified 23-item screening tool developed by the World Health Organization to examine lifetime and recent (past three months) substance use. Most sensitive item "*Have you ever used any drug by injection (NON-MEDICAL USE ONLY)?*"

To assess baseline alcohol and substance use problems, participants will complete the Short Inventory of Problems – Alcohol/Drug (SIP-AD) which measures negative consequences from drug or alcohol use. Most sensitive item "*I have taken foolish risks when I have been drinking/using drugs.*"

Depression, Physical and Mental Health. Two screening measures will be used to assess covariates and potential moderators (depression, physical health, mental health) of PARS outcomes. The two-item Patient Health Questionnaire (PHQ-2) assesses recent depressive symptoms. The most sensitive item is *"Over the last 2 weeks, how often have you been bothered by any of the following problems? Feeling down, depressed, or hopeless" Not at all… several days… more than half the days…. Nearly every day."* The EQ-5D is a widely used, and psychometrically validated, self-administered measure of health status. Most sensitive item: *"I have severe problems washing or dressing myself"*

Counselor Only Measures:

In order to assess a counselor's confidence in discussing client suicidality, we have developed a four item measure, PARS Confidence in Suicide Prevention Measure. Items include *"I am confident in dealing with the needs of suicidal clients. ____" and rated on a 0-10 scale.*

PARS Acceptability. Counselors will complete the PARS Counselor Acceptability Scale, a 13-item survey that was developed in our pilot trial to measure acceptability, ease, and perceived effectiveness of incorporating PARS into day-to-day IOP procedures. Items are measured on a Likert scale from strongly agree to strongly disagree. Most sensitive item: *"In general, the prevention program described was an intrusive procedure"*

Measures for Administrative Supplement:

[redacted]

[redacted]

5.14 Send HSD a Confidentiality Agreement if you will obtain or use any private identifiable UW records without subject's written consent (for example, screening medical records or class grades to identify possible subjects).

The Confidentiality Agreement form must be completed, printed, signed, and mailed to the Human Subjects Division at Box 359470. Your IRB application cannot be approved until we receive the Confidentiality Agreement.

6 CHILDREN (MINORS) and PARENTAL PERMISSION

6.1 Involvement of minors. Does your research include minors (children)?

Minor or child means someone who has not yet attained the legal age for consent for the research procedures, as described in the applicable laws of the jurisdiction in which the research will be conducted. This may or may not be the same as the definition used by funding agencies such as the National Institutes of Health.

- In Washington State the generic age of consent is 18, meaning that anyone under the age of 18 is considered a child.
- There are some procedures for which the age of consent is much lower in Washington State. See the WORKSHEET: Children for details.
- The generic age of consent may be different in other states, and in other countries.

X	No	→ If no, go to Section 8.
	Yes	→ If yes, provide the age range of the minor subjects for this study and the legal age for consent in your population(s). If there is more than one answer, explain.

N/A

	Don't know	→This means is it not possible to know the age of your subjects. For example, this may be true for some research involving social media, the Internet, or a dataset that you obtain from another researcher or from a government agency. Go to Section 8.

7.5 Children who reach the legal age of consent during participation in longitudinal research.

Children who were enrolled at a young age and continue for many years: It is best practice to re-obtain assent (or to obtain it for the first time, if you did not at the beginning of their participation).
Children who reach the legal age of consent: You must obtain informed consent from the now-adult subject for (1) any ongoing interactions or interventions with the subjects, or (2) the continued analysis of specimens or data for which the subject's identify is readily identifiable to the researcher, unless the IRB waives this requirement.

a. Describe your plans (if any) to re-obtain assent from children.

N/A

b. Describe your plans (if any) to obtain consent for children who reach the legal age of consent.

- If you plan to obtain consent, describe what you will do about now-adult subjects whom you are unable to contact.
- If you do not plan to obtain consent or think that you will be unable to do so, explain why.

N/A

7.6 Other regulatory requirements. (This is for your information only; no answer or response is required.)
Researchers are responsible for determining whether their research conducted in schools, with student records, or over the Internet comply with permission, consent, and inspection requirements of the following federal regulations:

- PPRA – Protection of Pupil Rights Amendment
- FERPA – Family Education Rights and Privacy Act
- COPPA – Children's Online Privacy Protection Act

8 CONSENT OF ADULTS

Review the following definitions before answering the questions in this section.

**CONSENT**	is the process of informing potential subjects about the research and asking them whether they want to participate. It usually (but not always) includes an opportunity for subjects to ask questions. It does not necessarily include the signing of a consent form. This question is about the consent process.
**CONSENT DOCUMENTATION**	refers to how a subject's decision to participate in the research is documented. This is typically obtained by having the subject sign a consent form.
**CONSENT FORM**	is a document signed by subjects, by which they agree to participate in the research as described in the consent form and in the consent process.
**ELEMENTS OF CONSENT**	are specific information that is required to be provided to subjects.
**PARENTAL PERMISSION**	is the parent's active permission for the child to participate in the research. Parental permission is subject to the same requirements as consent, including written documentation of permission and required elements.

**SHORT FORM CONSENT**	is an alternative way of obtaining written documentation of consent that is most commonly used with individuals who are illiterate or whose language is one for which translated consent forms are not available.
**WAIVER OF CONSENT**	means there is IRB approval for not obtaining consent or for not including some of the elements of consent in the consent process.
**WAIVER OF DOCUMENTATION OF CONSENT**	means that there is IRB approval for not obtaining written documentation of consent.

8.1 Groups Identify the groups to which your answers in this section apply.

- [X] Adult subjects
- [ ] Parents who are providing permission for their children to participate in research

→ *If you selected **PARENTS**, the word "consent" below should also be interpreted as applying to parental permission and "subjects" should also be interpreted as applying to the parents.*

8.2 The consent process. This series of questions is about whether you will obtain consent for all procedures except recruiting and screening and, if yes, how.

The issue of consent for recruiting and screening activities is addressed in question 4.6. You do not need to repeat your answer to question 4.6.

a. Are there any procedures for which you will not obtain consent?

- [X] No
- [ ] Yes → If yes, use the table below to identify the procedures for which you will not obtain consent. "All" is an acceptable answer for some studies.

Be sure to consider all research procedures and plans, including future contact, and sharing/banking of data and specimens for future work.

Group[1]	Describe the procedures or data/specimen collection (if any) for which there will be NO consent process	Reason why you will not obtain consent	Will you provide subjects with info about the research after they finish? YES	NO
			☐	☐
			☐	☐
			☐	☐
			☐	☐
			☐	☐

Table footnotes

1. *If your answer is the same for all groups you can collapse your answer across the groups and/or procedures.*

b. **Describe the consent process, if you will obtain consent for any or all procedures, for any or all groups. Address groups and procedures separately if the consent processes are different.**

Be sure to include:

- *The location/setting where consent will be obtained*
- *Who will obtain consent (refer to positions, roles, or titles, not names).*
- *Whether/how you will provide an opportunity for questions*
- *How you will provide an adequate opportunity for the subjects to consider all options*

Client Participants
Research staff will describe the study to the clients after their treatment group (or at a different time, if requested by a potential participant). Uninterested clients will then leave. There are three consent forms (a) the main consent, (b) follow-up locating consent form and (c) release of information form for their agency to release the medical record to the study. Research staff will distribute and review the consent forms with all interested clients. Clients will then be directed to review the consent forms individually and ask any questions they have about the study. Potential participants willing to participate will be asked to sign the consent form and then complete Contact Information Form and gift certificate reimbursement information.

Counselor Participants
Research staff will describe the study to the counselors. Uninterested counselors will then leave. Research staff will distribute and review the consent form with all interested counselors. Counselors will then be directed to review the consent form individually and ask any questions they have about the study. Potential participants willing to participate will be asked to sign the consent form, provide an email or phone number to be texted or called for follow-up assessments, and the gift certificate reimbursement information.

If either client or counselor participants do not want their agency to know that they did not want to participate, they will be told they are free to write random words on the consent form and/or questionnaires so no one else will know they were not participating.

Our goal is to attempt recruitment of all counselors at each site at each Step of the study. At Steps 2-6, the study will reach out to the site's point of contact to determine if there are any counselors who have left or new counselors hired since the previous Step. New counselors can then be recruited. To prevent inadvertent re-recruitment of counselors who refused to participate at an earlier Step, we will retain their names only in our records so that the research staff can provide a complete list of counselors from the previous Step to the site point of contact and clarify who refused so they won't be re-recruited.

Follow-up Locating Consent Form to Prevent Client Participant Attrition.
Our goal is for all participants to complete all outcome assessments regardless of treatment participation. Therefore, informed consent will include a separate locating consent form on which client participants choose locating strategies they consent to have used (e.g., obtaining forwarding address from post office, checking social media) and provide alternative contacts to whom the study can reach out in case the participant moves or changes contact information.

This form is separate from the main consent so that if we have to provide to a contact so they know we have permission to get the client's contact information, we are not sharing any information about the study, their drug use, etc. We have used this locating consent form for over 20 years of research. It finds an excellent balance between obtaining detailed information and allowing participants to only provide information they are comfortable providing.

Administrative Supplement

[redacted]

[redacted]

[redacted]

c. <u>Comprehension</u>. Describe how you will ensure or test the subjects' understanding of the information during the consent process.

The main points of the consent form will be reviewed with opportunities to ask question before the participants are asked to review and sign the informed consent form to assure that they understand.

d. <u>Influence</u>. Does your research involve any subject groups that might find it difficult to say "no" to your research because of the setting or their relationship with you, even if you don't pressure them to participate?

Examples: Student participants being recruited into their teacher's research; patients being recruited into their healthcare provider's research, study team members who are participants; outpatients recruited from an outpatient surgery waiting room just prior to their surgery.

	No
X	Yes

→ If yes, describe what you will do, for each of these subject groups, to reduce any effect of the setting or relationship on their decision.

Examples: a study coordinator will obtain consent instead of the subjects' physician; the researcher will not know which subjects agreed to participate; subjects will have two days to decide after hearing about the study.

All client participants will be asked by their counselors at the end of a standard treatment group in their IOP if they would like to volunteer for a study that involves staying after that group (or participating before or after one of the other IOP groups that week, if that day is not convenient). Client participants will be told by the agency and the study that this study is voluntary and will not affect their treatment nor will any data provided be shared with the agency unless the client reports suicide risk in which case the study will coordinate with the client and agency to assure adequate care. As noted above, if either clients or counselors do not want to participate, they can write nonsense on the forms so others do not know they are declining.

See 8.2b above for how this is addressed for the administrative supplement.

e. Ongoing process. For research that involves multiple or continued interaction with subjects over time, describe the opportunities (if any) you will give subjects to ask questions or to change their minds about participating.

Participants are free to stop participating any time as described in the consent form. If a participant no longer wishes to participate, they can decline when we contact them for follow-up assessments. We will ask them to elaborate on why they would like to discontinue participation in the study as this will help with current and future retention, but they are free to say that it is for no reason or they do not want to give a reason.

8.3 Written documentation of consent. Which of the statements below describe whether you will obtain documentation of consent? NOTE: This question does not apply to screening and recruiting procedures which have already been addressed in question 4.6.

Documentation of consent that is obtained electronically is not considered written consent unless it is obtained by a method that allows verification of the individual's signature. In other words, saying "yes" by email is rarely considered to be written documentation of consent

a. Are you obtaining written documentation of consent for:

- [ ] None of your research procedures → Use the table below to provide your justification then go to question 8.4.
- [ ] All of your research procedures → Do not complete the table; go to question 8.3.b.
- [X] Some of your research procedures → Use the table below to identify the procedures for which you will not obtain written documentation of consent from your adult subjects.

Adult subject group[1]	Describe the procedures or data/specimen collection (if any) for which there will be NO documentation of consent	Will you provide them with a written statement describing the research (optional)? YES	NO
Counselor Participants	We have decided that the study Steps will be 4 months in duration instead of 6 months as is currently approved.	X	
Counselor Participants	We have decided to add a final (7th) assessment for Counselor participants to allow us to determine the impact of Step 6.	X	
	Also see 'Procedure Stage' in Section 5.1		

[redacted]	[redacted]	X	☐
		☐	☐

Table footnotes

1. *If your answer is the same for all adult groups or all procedures, you can collapse your answer across the groups and/or procedures.*

8.4 Non-English-speaking or -reading adult subjects. Will you enroll adult subjects who do not speak English or who lack fluency or literacy in English?

[X] No

[ ] Yes → If yes, describe the process you will use to ensure that the oral and written information provided to them during the consent process and throughout the study will be in a language readily understandable to them and (for written materials such as consent forms or questionnaires) at an appropriate reading/comprehension level.

N/A

a. Interpretation. Describe how you will provide interpretation and when. Also, describe the qualifications of the interpreter(s) – for example, background, experience, language proficiency in English and in the other language, certification, other credentials, familiarity with the research-related vocabulary in English and the target language.

N/A

b. Translations. Describe how you will obtain translations of all study materials (not just consent forms) and how you will ensure that the translations meet the UW IRB's requirement that translated documents will be linguistically accurate, at an appropriate reading level for the participant population, and culturally sensitive for the locale in which they will be used.

N/A

8.5 Barriers to written documentation of consent. There are many possible barriers to obtaining written documentation of consent. Consider, for example, individuals who are functionally illiterate; do not read English well; or have sensory or motor impairments that may impede the ability to read and sign a consent form.

a. Describe your plans (if any) for obtaining written documentation of consent from potential subjects who may have difficulty with the standard documentation process (that is, reading and signing a consent form). Skip this question if you are not obtaining written documentation of consent for any part of your research.

Examples of solutions: Translated consent forms; use of the Short Form consent process; reading the form to the person; excluding individuals who cannot read and understand the consent form.

If a participant is unable to read a consent, due to visual impairment, study staff will read the consent to the participant. We will have the participant sign to the best of their ability, and then printed by the research

study team. We will also have a witness sign the consent for added precaution. All of this will be clearly documented within our tracking systems to ensure clarity.

8.6 Deception. Will you deliberately withhold information or provide false information to any of the subjects?

[X] **No**
[ ] **Yes** → If yes, describe what information and why.

Example: you may wish to deceive subjects about the purpose of the study.

N/A

a. Will you debrief the subjects later? (Note: this is not required.)

[ ] **No**
[ ] **Yes** → If yes, describe how you will debrief the subjects. Upload any debriefing materials, including talking points or a script, to the **Consent Form and Recruitment Materials** SmartForm of ***Zipline***.

N/A

8.7 Cognitively impaired adults, and other adults unable to consent.

a. Cognitively impaired adults and other adults unable to consent. Do you plan to include such individuals in your research?

Examples: individuals with Traumatic Brain Injury (TBI) or dementia; individuals who are unconscious, or who are significantly intoxicated.

[X] **No** → If no, go to question 8.8.
[ ] **Yes** → If yes, answer the following questions.

a.1. Rationale. Provide your rationale for including this population in your research.

N/A

a.2. Capacity for consent / decision making capacity. Describe the process you will use to determine whether a cognitively impaired individual is capable of consent decision making with respect to your research protocol and setting. If you will have repeated interactions with the impaired subjects over a time period when cognitive capacity could increase or diminish, also describe how (if at all) you will re-assess decision-making capacity and consent during that time.

N/A

a.3. Permission (surrogate consent). If you will include adults who cannot consent for themselves, describe your process for obtaining permission ("surrogate consent") from a legally authorized representative (LAR).

*For research conducted in Washington State, see the **SOP: Legally Authorized Representative** to learn which individuals meet the state definition of "legally authorized representative".*

N/A

a.4. Assent. Describe whether assent will be required of all, some, or none of the subjects. If some, indicate which subjects will be required to assent and which will not (and why not). Describe any process you will use to obtain and document assent from the subjects.

N/A

a.5. Dissent or resistance. Describe how you will identify the subject's objection or resistance to participation (including non-verbal) during the research, and what you will do in response.

N/A

8.8 Consent-related materials. Upload to the **Consent Forms and Recruitment Materials** SmartForm of ***Zipline*** all consent scripts/talking points, consent forms, debriefing statements, Information Statements, Short Form consent forms, parental permission forms, and any other consent-related materials you will use.

- *Translations must be included. However, you are strongly encouraged to wait to provide them until you know that the IRB will approve the English versions.*
- *Combination forms: It may be appropriate to combine parental permission with consent, if parents are subjects as well as providing permission for the participation of their children. Similarly, a consent form may be appropriately considered an assent form for older children.*
- *For materials that cannot be uploaded: upload screenshots or written descriptions that are sufficient to enable the IRB to understand the types of data that will be collected and the nature of the experience for the participant. You may also provide URLs (website addresses) or written descriptions below. Examples of materials that usually cannot be uploaded: mobile apps; computer-administered test; licensed and restricted standardized tests.*

9 PRIVACY AND CONFIDENTIALITY

9.1 Privacy protections. Describe the steps you will take, if any, to address possible privacy concerns of subjects and potential subjects.

Privacy refers to the sense of being in control of access that others have to ourselves. This can be an issue with respect to recruiting, consenting, sensitivity of the data being collected, and the method of data collection.
Examples:

- *Many subjects will feel a violation of privacy if they receive a letter asking them to participate in a study because they have ____ medical condition, when their name, contact information, and medical condition were drawn from medical records without their consent. Example: the IRB expects that "cold call" recruitment letters will inform the subject about how their information was obtained.*
- *Recruiting subjects immediately prior to a sensitive or invasive procedures (e.g., in an outpatient surgery waiting room) will feel like an invasion of privacy to some individuals.*
- *Asking subjects about sensitive topics (e.g. details about sexual behavior) may feel like an invasion of privacy to some individuals.*

Questionnaires with a blank cover sheet will be provided. Client participants will be told by the agency and the study that this study is voluntary and will not affect their treatment nor will any data provided or shared with the agency unless the client reports suicide risk, in which case the study will coordinate with the client and agency to assure adequate care. Counselor participants will be told by the agency and the study that this study is voluntary and participation, or non-participation, will not affect their job nor will any data provided be shared with the agency.

If a client participant is not considered appropriate to participate in these procedures, the IOP counselor will call that client aside as the group ends to explain this to them privately and they will not stay to hear about the study or participate.

The primary risks in this study are discomfort with answering questions about suicidality or drug use and a violation of confidentiality. The latter could occur from someone seeing the individual's answers during the group session, seeing the text messages, emails, or online survey at follow-up, or mistakes in data security by the research team.

Should a client express distress about talking about suicide in the PARS intervention, this will be addressed by the counselor leading the group as they would in other IOP sessions (in which many difficult topics including trauma, depression, and sexually transmitted diseases are standard topics). There will be one exception due to the potential for suicide risk. Following group, the counselor will meet individually with the client to address the issue and conduct a suicide risk assessment. Should the client be found to be at risk, standard IOP protocols for suicide risk will be followed by the counselor. Refer to Section 10 for more detail.

Participants are not required to answer any assessment question they choose not or don't know how to answer. This is described in the consent form and the follow-up survey protocol will include a method to opt out if they do not want to answer a specific item. Participants are free to stop at any time and the amount of money per question is low enough that we believe it will not be coercive if they do not want to participate.

All participants are adults and voluntarily participating, but we recognize that clients in addiction treatment and counselors might have specific or idiosyncratic reasons why participation in this study is inadvisable. Therefore, we have included an exclusion criterion for each to exclude anyone the agency or research team believes should not participate. This will be handled subtly, without public announcement that they should not participate, to protect their privacy (e.g., a client is called over by a counselor or a counselor is called over to chat with an agency leader in an innocuous way at the end of the meeting where we were recruiting and taken from public view before telling them that they believe the study is not a fit for them.)

9.2 Identification of individuals in publications and presentations. Do you plan to use potentially identifiable information about subjects in publications and presentations, or is it possible that individual identities could be inferred from what you plan to publish or present?

- [X] **No**
- [ ] **Yes** → If yes, will you obtain subject consent for this use?
  - [ ] **Yes**
  - [ ] **No** → If no, describe the steps you will take to protect subjects (or small groups of subjects) from being identifiable.

N/A

10.2 Reproductive risks. Are there any risks of the study procedures to men and women (who are subjects, or partner of subjects) related to pregnancy, fertility, lactation or effects on a fetus or neonate?

Examples: direct teratogenic effects; possible germline effects; effects on fertility; effects on a woman's ability to continue a pregnancy; effects on future pregnancies.

[X] **No** → If no go to question 10.3
[ ] **Yes** → If yes, answer the following questions:

a. Risks. Describe the magnitude, probability, duration and/or reversibility of the risks.

N/A

b. Steps to minimize risk. Describe the specific steps you will take to minimize the magnitude, probability, or duration of these risks.

Examples: inform the subjects about the risks and how to minimize them; require a pregnancy test before and during the study; require subjects to use contraception; advise subjects about banking of sperm and ova.

If you will require the use of contraception: describe the allowable methods and the time period when contraception must be used.

N/A

c. Pregnancy. Describe what you will do if a subject (or a subject's partner) becomes pregnant

For example; will you require the subject to immediately notify you, so that you can discontinue or modify the study procedures, discuss the risks, and/or provide referrals or counseling?

N/A

10.3 Unforeseeable risks. Are there any research procedures that may have risks that are currently unforeseeable?

Example: using a drug that hasn't been used before in this subject population.

[ ] **No**
[X] **Yes** → If yes, identify the procedures.

Although not adverse event, a possible problem is the discovery of suicidality that was previously unknown. In this case, we will facilitate referral back to agency or other treatment options as fits the client's needs using the risk assessment protocol as noted above in 9.3. No other adverse events are expected, but if they occur the participant will be referred back to their agency or other options.

While the discovery of suicidality is not an adverse event, the research team will respond according to our standard suicide risk management protocol. This research team has considerable experience in managing suicidal individuals in study assessments and Drs. Comtois or Ries or Ms. Kerbrat who is a licensed social worker will be available during all baseline assessments.

The research team who is conducting a large clinical trials of suicidal Service Members using text messages through the REDCap platform, the team already has an alert and follow-up system in place to assure immediate response if responses indicating suicide risk occur. When alerts go off, the research team has an alert and back-up system to respond to these messages promptly and use the risk management plan from 9.3 to connect the participant to care.

10.4 Subjects who will be under regional or general anesthesiology. Will any research procedures occur while subjects-patients are under general or regional anesthesia, or during the 3 hours preceding general or regional anesthesia (supplied for non-research reasons)?

- [X] No
- [ ] Yes → If yes, check all the boxes that apply.
  - [ ] Administration of any drug for research purposes
  - [ ] Inserting an intra-venous (central or peripheral) or intra-arterial line for research purposes
  - [ ] Obtaining samples of blood, urine, bone marrow or cerebrospinal fluid for research purposes
  - [ ] Obtaining a research sample from tissue or organs that would not otherwise be removed during surgery
  - [ ] Administration of a radio-isotope for research purposes**
  - [ ] Implantation of an experimental device
  - [ ] Other manipulations or procedures performed solely for research purposes (e.g., experimental liver dialysis, experimental brain stimulation)

If you checked any of the boxes:
You must provide the name and institutional affiliation of a physician anesthesiologist who is a member of your research team or who will serve as a safety consultant about the interactions between your research procedures and the general or regional anesthesia of the subject-patients. If your procedures will be performed at a UW Medicine facility or affiliate, the anesthesiologist must be a UW faculty member.

N/A

*** If you checked the box about radio-isotopes: you are responsible for informing in advance all appropriate clinical personnel (e.g., nurses, technicians, anesthesiologists, surgeons) about the administration and use of the radio-isotope, to ensure that any personal safety issues (e.g., pregnancy) can be appropriately addressed. This is a condition of IRB approval.*

10.5 Data and Safety Monitoring. A Data and Safety Monitoring Plan (DSMP) is required for clinical trials (as defined by NIH). If required for your research, upload your DSMP to the **Supporting Documents** SmartForm in ***Zipline***. If it is embedded in another document you are uploading (for example, a Study Protocol, use the text box below to name the document that has the DSMP.

Please refer to supporting document.

A Data and Safety Monitoring Board (DSMB) will serve as an oversight committee, reviewing any modifications to the research design and conduct of the study and making recommendations according to the NIH Policies for data and safety monitoring as described below. The DSMB is charged as an external, independent oversight board, to monitor the conduct of the study for ongoing feasibility, data integrity, and safety. The specific goals of this DSMB are to:

1. Review new or modified risk management protocols at both sites.
2. Review procedures and decisions regarding the adequate protection of specific patients when investigators move into risk management protocols because of adverse events.

3. Review progress toward meeting enrollment goals.
4. Review procedures for maintaining the confidentiality of data, and quality of data collection, management, and analyses.
5. When appropriate, serve as final arbiters of whether individual patients should be removed from a protocol.
6. Recommend continuation, discontinuation, modification, or termination of a study based on emerging data (in the study and literature) and evaluation of risk/benefit ratio.
7. Conduct annual reviews to determine whether patient safety has been adequately safeguarded.
8. Meet at least once yearly with the principal investigator to review progress reports and more often as needed if severe adverse events occur which require discussion or changes to protocol.
9. Submit a brief report to NIH yearly summarizing the board's review.

Dr. Comtois has served on the NIMH DSMB-B for multi-site contracts since 2003 and has chaired and participated in multiple DSMBs for other studies of suicidal individuals. She has DSMBs for both of her current clinical trials. We have developed procedures based on this experience that are rigorous but feasible which we will follow in this study. Dr. Comtois will select DSMB members who have appropriate expertise, including in ethics and biostatistics, and no conflicts of interest. These members will monitor the study and ensure that monitoring is timely and effective.

Prior to the start of the study, the DSMB will review the IRB-approved protocol and overall plans for data and safety monitoring and request the desired data and format for reports from the study. The board will then review patient flow and participant entry to ensure adequate recruitment and retention of participants and will monitor the occurrence of adverse events related to participation in the protocol. If a severe adverse event is discovered, it will be communicated to the University IRB as well as the DSMB. The DSMB chair will determine if an immediate meeting to discuss a severe adverse event is needed. If so, a teleconference will be convened by Dr. Comtois. Otherwise, these events will be reviewed at the next scheduled meeting.

[redacted]

10.6 Un-blinding. If this is a double-blinded or single-blinded study in which the participant and/or you do not know the group to which the participant is assigned: describe the circumstances under which un-blinding would be necessary, and to whom the un-blinded information would be provided.

This study does not blind participants from knowing their treatment condition. We do not make telling them an explicit Step in the study (to reduce expectancies that might bias responses) but will tell anyone who asks at any time during the study.

10.7 Withdrawal of participants. If applicable, describe the anticipated circumstances under which participants will be withdrawn from the research without their consent. Also, describe any procedures for orderly withdrawal of a participant, regardless of the reason, including whether it will involve partial withdrawal from procedures and any intervention but continued data collection or long-term follow-up.

We do not anticipate a reason to withdraw participants without their consent. Should this be considered, Drs. Comtois and Ries will consult with each other and our treatment partners to determine why such a move is in the best interest of the client and only withdraw them for that reason. The DSMB Chair would be immediately informed as per DSMB protocol.

10.8 Anticipated direct benefits to participants. If there are any direct research-related benefits that some or all individual participants are likely to experience from taking part in the research, describe them below:

Do not include benefits to society or others, and do not include subject payment (if any). Examples: medical benefits such as laboratory tests (if subjects receive the results); psychological resources made available to participants; training or education that is provided.

The minimal risks to patient-subjects are reasonable in relation to the anticipated benefits because participants in the PARS condition will receive potentially valuable information regarding the relationship between addiction and suicide and what to do if suicide symptoms are seen in themselves or others. There is no anticipated benefit to participants in the TAU condition beyond receiving reimbursement for study participation.

10.9 Individual subjects findings.

a. Is it likely that your research will unintentionally discover a previously unknown condition such as a disease, suicidal intentions, or genetic predisposition?

[ ] **No**
[X] **Yes** → If yes, explain whether and how you would share the information with the subject.

It is possible that suicidality will be uncovered in study assessments or during the PARS intervention that were previously unknown to the treatment team. If this occurs during study assessments, the RA with assistance, as needed, from a licensed member of the research team will intervene as described in section 9.3 and will follow that protocol to assure adequate care for the individual while minimizing disclosure of study information to their treatment team to what they choose to tell. Drs. Comtois and Ries will train and supervise the RAs and intervene as needed to assure an appropriate response. If suicidality is disclosed during the PARS intervention, it will be to the counselor administering the PARS intervention who will follow standard agency procedures to intervene in the face of risk.

b. Do you plan to routinely share the individual results of your study procedures with the subjects – such as genetic test results, laboratory tests, etc.?

[X] **No**
[ ] **Yes** → If yes, complete and upload the SUPPLEMENT: Participant Results Sharing to the **Supporting Documents** SmartForm of ***Zipline***

10.10 Commercial products or patents. If a commercial product or patent could result from this study, describe whether subjects might receive any remuneration/compensation and, if yes, how the amount will be determined:

N/A

Specific Aims

The goal of this study is to evaluate the effectiveness and utility of our NIDA R21 developed "Preventing Addiction Related Suicide" (PARS) program[1] by utilizing a novel stepped wedge design to evaluate PARS as a selected prevention program to increase help-seeking by clients in community addiction treatment. Studies consistently show suicide and suicidal behaviors are highly related to substance use disorders (SUDs). Recent reviews find that the **risk of suicide is 10-17 times higher for people using multiple drugs, injecting drugs, and for alcohol use disorders.**[2,3] SUDs are also related to suicidal thoughts and suicide attempts. Clients admitted for alcohol treatment report a much higher rate of lifetime suicide attempts (40-43%) than a nationally representative sample of adults (4.6%).[4–6] Further, prospective data shows that **individuals in addiction treatment had five times the odds of suicide attempt over five years compared to those not in treatment**,[7] emphasizing addiction treatment as a key opportunity for instituting suicide prevention strategies.[8]

Based on Stage I guidelines for developing and adapting behavioral interventions[9,10] and information from a Substance Abuse and Mental Health Services Administration (SAMHSA) Treatment Improvement Protocol (TIP50) on suicide and addiction,[11] we developed the Preventing Addiction Related Suicide (PARS) program. To maximize the chances of implementation, PARS was developed to be a community-friendly program with a team of community partners (i.e., administrators, counselors, clients) who advised on its scope, duration, and approach. Community leaders reviewed PARS throughout its development and pilot testing was conducted in their community treatment settings. Thus, PARS is simultaneously based on evidence-based practice **and** the goals and needs of community treatment settings. Importantly, PARS is a selected prevention program and <u>not</u> intervention for suicidality *per se*—it is designed for all clients in addiction treatment as a standard part of care. PARS' goal is increased help-seeking by addiction treatment clients as well as by clients' friends and family if and when they themselves become suicidal. Reaching out for help leads to care that can address and resolve suicidality. **PARS is the only published selected prevention program for this high-risk population.**

PARS is a psychoeducational program taught as a single three-hour module integrated into a standard group therapy-oriented Intensive Outpatient Program (IOP), the most common form of community addiction treatment.[12] Pilot testing of PARS in three community agencies demonstrated significant post-intervention increases in accurate information about suicide and decreases in maladaptive attitudes toward suicide. These changes at post-intervention were maintained at 1-month follow-up. Even more compelling, 1-month follow-up assessments demonstrated that the likelihood of positive help seeking for suicidality doubled for the month after PARS compared to the month before. Clients were significantly more likely to ask suicidal friends (from 9% to 22%) and family (9% to 17%) to seek help as well as to seek help themselves (4% to 9%).

Given these promising Stage I results in Stage III settings, we propose a fully-powered Stage III effectiveness trial of PARS compared to Treatment-as-Usual (TAU) using a stepped wedge design with 900 clients enrolled in 15 community addiction treatment sites (see Figure 1 for design model). We will collect outcome data post-intervention and at 1, 3, and 6 months follow-up. We propose the following research aims:

Aim 1: Compare the effectiveness of IOP integrating PARS to TAU to change beliefs about suicide and suicide prevention.

Hypothesis 1a: Clients who receive PARS will know more accurate information about suicide

Hypothesis 1b: Clients who receive PARS will have less maladaptive attitudes about suicide

Aim 2: Compare the effectiveness of IOP integrating PARS to TAU to increase help-seeking behaviors for clients and for clients' friends or family at risk of suicide.

Hypothesis 1c: Clients who receive PARS will show greater help-seeking for themselves and others

Aim 3: Evaluate whether changes in beliefs about suicide and suicide prevention—particularly regarding warning signs for suicide, including addiction, intoxication, and relapse, as well as beliefs that suicide is preventable when action is taken—are possible mechanisms by which PARS increases help-seeking behavior.

Hypothesis 2: The effect of PARS vs. TAU on changes in help-seeking will be mediated by improved information and attitudes

Exploratory Aim 4: Evaluate possible clinic-level dose effects of PARS administration such that participant outcomes improve the longer PARS is implemented within clinics.

Exploratory Aim 5: Compare the effects of PARS vs. TAU on clients' suicidality *and substance use* in the follow-up period.

By integrating PARS into IOP group treatment, community treatment agencies are in a unique position to act as key players in the national suicide prevention strategy by providing suicide prevention information, improving attitudes regarding suicide, and increasing help-seeking skills for one of the most high-risk populations for suicide. This proposal is innovative in its focus, the development of PARS in community settings, as well as the use of a stepped wedge design.

A. Background and Significance

1. Suicidal behavior is prevalent and costly in substance-abusing populations.

Suicide and suicidal behaviors are over-represented in populations with substance use disorders (SUDs) compared to the general adult population. Recent reviews find that the risk of suicide is 14 times higher for people injecting drugs, 10 times for alcohol use disorders, and 17 times for polydrug users.[2,3] Clients receiving alcohol treatment are about 10 times more likely to endorse of a lifetime history of suicide attempts (43%)[4,5] compared to a nationally representative sample of adults (4.6%).[6] Moreover, prospective data shows that individuals in addiction treatment had five times the odds of suicide attempt over five years compared to those not in treatment.[7] Suicidal behavior places a heavy burden on the health services system, resulting in more than 650,000 hospital visits and $2 billion in health care costs each year, while suicide deaths result in an annual economic burden of $44 billion.[13,14] Thus, consistent with NIDA's mission to identify and test population-level approaches for the prevention of drug-related problems, there is a need to develop and evaluate effective behavioral treatments that can be easily and widely implemented to reduce suicidal behaviors in substance-abusing populations. PARS is a prime candidate for such research, as it has shown promising results in Stage I research (i.e., intervention generation and refinement, pilot testing, and feasibility) conducted in community treatment settings (R21 DA026494). Funding the proposed Stage III research (i.e., "efficacy in the real world") trial would further evaluate PARS' effectiveness and utility in community settings.

2. Community addiction treatment is an ideal setting for targeting suicide risk in this high-risk group.

Every year, approximately 2.5 million people in the United States enter specialized addiction treatment programs.[11] By far, the most common modality of publicly funded addiction treatment available is group-based Intensive Outpatient Programs (IOP).[12] Thus, adding evidence-based, transportable suicide prevention strategies into the standard IOP treatment package has the potential to reach an enormous number of people who are at very high risk for suicide. Moreover, entering addiction treatment may represent a key window for intervention to reduce suicidal behaviors, as this transition is marked by high rates of suicidal thinking and behavior. Individuals often enter addiction treatment in the context of multiple increased risk factors for suicide: when substance use is out of control and/or is resulting in particularly severe impairment (e.g., marital or financial difficulties, severe depressive symptoms).[15] Between 10% and 40% of clients entering addiction treatment report suicidal ideation with a plan.[16,17] Roughly one out of every 25 clients entering addiction treatment report having made a suicide attempt in the 30-day period before treatment,[18] while one in four report having made a suicide attempt in the past year.[19] Moreover, suicide risk is known to be highly fluid,[20] and although most IOPs will screen patients for suicide risk at the outset of treatment, suicidal ideation and behaviors are likely to fluctuate over the course of treatment, particularly if high-risk situations such as relapse occur during treatment.[11] Thus, addiction IOPs will frequently treat people who are or have recently been suicidal,[18] and have the potential to directly intervene to reduce these problems. Clients with addiction also connect with each other during treatment, in twelve-step meetings, and in drug use. Improving accurate information and adaptive attitudes toward suicide prevention as well as how to effectively reduce risk and reach out for help may not only increase their access to care if suicidal but also increase access of their friends and family who are often also at risk.

3. Addiction treatment providers need additional training to prevent suicidal behavior.

Unfortunately, most chemical dependency counselors feel unprepared, inadequately trained, and uncomfortable addressing the issue of suicide.[21,22] Recently, there have been several efforts to respond to this need. For example, SAMHSA recently developed the Treatment Improvement Protocol number 50 (TIP50),[11] which provides best-practice guidelines for counselors and program administrators to effectively assess, manage and respond to acute suicide risk within addiction treatment programs. The TIP50 is associated with significantly increased staff self-efficacy, suicide-related knowledge, and suicide prevention behaviors.[23] Moreover, some states (e.g., Washington and Kentucky) have recently passed legislation requiring suicide prevention training for all human services personnel, including addictions counselors.[24] While these efforts are good first steps, they primarily focus on managing suicidal crises (e.g., assessing risk and determining when to refer the client to a higher level of service). Instead of aiming to treat acutely suicidal people, PARS aims to deliver an upstream prevention program to an at-risk population. This selected suicide prevention program thus has the potential to serve a dual purpose of providing prevention for clients, and providing ongoing education and training for the addiction treatment staff tasked with delivering the program.

4. Built-in therapist adherence and fidelity to model.

One of the challenges of implementing suicide prevention programs in community treatment settings is the limited resources for ongoing training. PARS was developed to easily fit within the daily work and training models of community IOPs. PARS is delivered in a single IOP session (typically, a 3-hour group therapy

session) using a detailed PowerPoint presentation (developed by the R21) to provide structure, psychoeducational content, and defined discussion periods. Clients are taught to recognize the warning signs for suicide in themselves and others and how to respond effectively by seeking support and treatment. Pilot testing showed that naïve community addiction counselors could be trained to competently and adherently deliver PARS in a single three-hour training session that utilized the same set of PARS PowerPoints that counselors later used in an IOP session with addictions clients. In this way, the PARS training model promotes faithful administration of the program without unduly burdening clinical and administrative staff. Counselors rated this model of training as very acceptable (62-69% "strongly agreed" that PARS would be acceptable and appropriate to addictions counselors, and would be beneficial to clients). Moreover, by repeatedly delivering PARS with each new IOP group cohort, counselors will be re-exposed to suicide-relevant risk assessment and help-seeking strategies, potentially continuing to improve their comfort and competence in discussing and determining suicide risk, and intervening when appropriate.

5. A selected prevention approach can expand impact and reach.

Although the empirical base for interventions to reduce suicide has rapidly developed in the last 30 years, many of these interventions have either aimed at 1) reducing suicide rates in an entire populations (universal prevention, e.g., suicide awareness media campaigns), limiting our ability to detect immediate and short-term effectiveness, or 2) only provided to individuals already presenting with acute suicidality (indicated prevention, e.g., individual cognitive-behavioral therapy for substance users with suicidal ideation or recent attempts[25]), limiting their generalizability. In contrast to these approaches, a selected prevention strategy can have greater reach and impact by offering a compromise between these approaches. Selected prevention aims to intervene before suicidal thinking or behavior develops. Rather than targeting the entire population or the specific individual, selected prevention targets all individuals within a pre-selected high-risk population—in this case, adults presenting for addiction treatment. Because selected prevention assumes that not every individual receiving the program will experience the problem of interest (i.e., suicidal ideation or behavior), it aims to increase knowledge and attitudes that will facilitate help-seeking on behalf of the client as well as his or her peers, increasing the reach of PARS to include other individuals in the client's social network. Previous randomized controlled trials have shown that comparable, school based selected suicide prevention programs upon which PARS was based result in significantly increased rates of help-seeking on behalf of self and others[26], as well as lower rates of suicidal thoughts, threats and attempts in high school students.[26–29]

6. Changing behavior through changes in beliefs about suicide has been effective.

PARS was developed by adapting existing, empirically supported suicide prevention programs to fit substance use settings and populations. The three programs that informed PARS are: 1) Signs Of Suicide (SOS)[26,27], 2) Counselors Care, Assess, Respond, Empower (C-CARE)[28–30], and 3) Coping and Support Training (CAST).[28–30] Each of these programs aims to promote help seeking through two primary mechanisms. First, by providing education about warning signs for suicide, the programs are expected to increase recognition of depressive and suicide-related symptoms, which in turn is expected to promote help seeking. Second, by reducing stigma and promoting more adaptive attitudes toward suicide, the programs are expected to reduce barriers to help seeking. A broad definition of help seeking is encouraged, including not only referrals for a mental health professional but also seeking support from other resources (e.g., case managers, crisis lines, loved ones). Evidence from previous trials supports these mechanisms of change. For example, in a randomized controlled trial of the SOS program in high school students, reductions in suicide attempts in the treatment group were mediated by self-reported increases in suicide-related information and adaptive attitudes.[26] Specifically, more adaptive attitudes and more accurate knowledge of suicide risk factors were each uniquely and significantly related to lower probability of suicide attempts, and accounted for approximately 40% of the variance in treatment outcomes. An important next step is to examine whether a similar mechanism of change explains treatment effects in substance-using populations.

7. PARS was developed to be transportable, disseminable, and community-friendly.

PARS is a psychoeducational behavioral treatment that aims to increase knowledge, attitudes, and behaviors that can promote recognition of and help-seeking for suicide risk among clients receiving addiction treatment. PARS is taught as a single three-hour module that is integrated within standard IOP therapy group treatment. From its inception, PARS was developed as a community-friendly suicide prevention program that can be easily incorporated into existing addiction treatment programs and agencies, consistent with NIDA's strategic priorities. All feasibility testing of PARS was conducted in community treatment programs.[1] Prior funding (R21 DA026494) enabled Stage I treatment development research, during which existing behavioral suicide prevention treatments were adapted and modified for substance abusing populations (Stage IA), and pilot and feasibility testing was conducted (Stage IB). The proposed project will move this promising line of research to

Stage III by conducting a large-scale, experimental test of the effectiveness of PARS in real-world, community IOP settings. Moving from Stage I to Stage III is appropriate when the intervention was developed in community settings, when Stage I has produced promising findings as well as established methods to ensure fidelity of delivery and therapist training materials, and when promoting implementation is a major goal of the research. Consistent with NIDA's research priorities, our proposal includes explicit examination of the putative mechanisms of behavioral change that were highlighted in Stage I research. In sum, the proposed research takes a critical next step by evaluating the real-world efficacy of a behavioral treatment that has the potential to reduce a prevalent, important, and costly behavior that is a major cause of death for individuals with SUD.[31]

B. Innovation

1. **Innovative use of IOPs to reduce risk of suicide:** PARS is an innovative approach that allows addiction treatment agencies to act as key players who can reduce and possibly prevent suicide in one of the most high-risk for suicide populations in the USA.[8] To our knowledge, this proposal is one of the first studies to use a randomized design to examine the effectiveness of suicide prevention within addiction IOPs.
2. **Innovative dissemination and health services impact:** PARS is innovative in being developed and evaluated in "real-world" clinical sites that provide both public and private addiction services. PARS was designed based on clinical and administrative input to not only fit the recovery philosophy and clinical approach of IOP settings but also to fit into the billing and employee models of community addiction agencies. This provides high likelihood that PARS could be readily implemented, allowing PARS to reach millions of people who seek treatment every year,[11] should it prove to be effective.
3. **Use of innovative research design:** The goal of this Stage III trial is to evaluate the efficacy of PARS while being responsive to the unique challenges of conducting randomized, controlled research in real-world, community treatment settings. In line with Stage II trials, we strive for rigorous research methods that maintain adequate internal validity. This study is therefore unique in that it combines a rigorous, randomized design with a disseminable suicide prevention program that targets a very high-risk population. In striking a balance between these two goals, we propose to use a stepped wedge randomized trial design. This design allows for a sequential roll-out of PARS to all of the community agencies enrolled in this study by the end of the trial, but promotes internal validity by randomly assigning each site to a "step" which will determine the timing of its transition from control (i.e., TAU) to treatment (i.e., PARS). Thus, while each client experiences either PARS or TAU only once, clinics differ in their exposure to PARS based on the timing of their transition to PARS in the stepped wedge design. This design has a number of benefits over the traditional RCT. Like cluster randomization, it allows randomization at the site rather than individual level which fits with a Stage III evaluation of the intervention as implemented in community treatment. Furthermore, stepped wedge trials facilitate the examination of dose-response or delay effects at the clinic level by modelling the association between the time clinics spent in the PARS phase and the effectiveness of PARS on clients. Finally, a stepped wedge design facilitates complete implementation of PARS among our 15 sites, enhancing the clinical practice benefits of this trial for our community partners.
4. **Explicit examination of treatment outcome mediators:** Because the proposed research directly examines potential mediators of treatment effectiveness (i.e., accurate information and adaptive attitudes mediating improved help-seeking behavior), this study can guide future efforts to improve the PARS program. Identifying mechanisms of treatment action may allow us to refine PARS; making it more potent and targeted by increasing the emphasis on challenging maladaptive attitudes, increasing factual knowledge, or both.
5. **Built-in counselor re-training:** As noted above, PARS could represent an innovative training strategy for counselors who are consistently updated in these competencies by repeatedly using skills taught through PARS training with each new IOP group cohort. Few studies have examined the effects of repeated training exposure on suicide prevention effectiveness for clients, thus our exploratory aim examining dose effects may make an important contribution to this literature by testing whether there are effects of repeated exposure to PARS at the clinic level on client outcomes.

C. Approach

C.1. Preliminary Studies

R21 Preventing Addiction Related Suicide (PARS) study.

As part of a NIDA R21, a pre-post pilot study of PARS was conducted with clients attending group-based IOP addiction treatment at one of three publicly funded addiction treatment agencies in Washington State.[1] All agencies were members of the NIDA Clinical Trials Network (CTN) and were in urban areas. Prior to PARS, none of the agencies included a suicide module in their IOP programs (for more detail on IOP in Washington State, see Design section C.3). Seventy-nine clients were approached, of whom 78 consented to participate.

The inclusion criterion was current participation in IOP treatment. Exclusion from participation was based on the following criteria: (1) imminently suicidal patients as well as those who had planned or attempted suicide within the past 3 months, (2) patients with cognitive or language barriers judged severe enough to impede participation (no one was excluded on either criteria). Follow-up data were collected from 64 patients at the 1-month time point, for an 82% follow-up rate. Patients were given a $25 gift card for completing each survey.

The mean age of the total patient sample was 35 years old (SD=1.20), 64% of which were male. While the modal level of education was a high school diploma (58%), an additional 17% did not complete high school education or the equivalent. Almost half (44%) of the sample was Caucasian, 26% were African American, 8% Asian, 5% American Indian/Alaskan Native, 6% more than one race, and 8% did not report race.

Thirteen IOP counselors from the three agencies described above were recruited and trained to administer PARS. After completing informed consent and receiving training, counselors answered a survey about PARS acceptability and utility in the standard working conditions at their sites. The average age of the participating counselors was 46 years and the average length of time working in the addiction field was 5.6 years with a range of 6 months to over 20 years. Eight of the 13 counselors were female (62%) and 12 were Caucasian (92%). The modal level of education was a bachelor's degree, and all had state certified chemical dependency counseling credentials as required by their agencies and Washington State.

PARS was administered as described below and in Voss et al.[1] Although none of the pilot participants reported imminent risk of suicide, several reported suicide attempts more than 3 months ago and loss of family and friends to suicide, which led to meaningful group discussion among clients during the PARS session.

As can be seen in Table 1, this pilot study of PARS demonstrated significant post-intervention increases in accurate information and decreases in maladaptive attitudes toward suicide among client participants. Significant gains compared to pre-intervention were maintained at 1-month follow-up for both information and maladaptive attitudes. Help-seeking was also significantly improved. Compared to the month before PARS, in the month following PARS, pilot participants were twice as likely to ask friends and family to seek help as well as to seek help themselves. This also highlights the fluidity of suicide risk during addiction treatment—although outpatient SUD treatment providers attempt to screen out acutely suicidal individuals at intake, instead referring them to a higher level of care, this is nonetheless a high-risk population and suicidal thoughts and behavior are not uncommon during community addiction treatment.

**Table 1 R21 Pilot Study Results**	Pre-Test	Post-Test	One Month
Accurate information about suicide (scores range from 0-14)	5.3	9.4^{***}	8.1^{***}
Maladaptive attitudes toward suicide (scores range from 9-45)	19.3	15.6^{***}	17.3^{***}
Asked friend to get help with suicidal thoughts/feelings in past month	9%	-	$22\%^{**}$
Asked family to get help with suicidal thoughts/feelings in past month	9%	-	$17\%^{*}$
Asked for help him/herself with suicidal thoughts/feelings in past month	4%	-	$9\%^{**}$
Any help-seeking in past month	15.6%	-	28.1%

$^{*}p < .05$ $^{**}p < .01$ $^{***}p < .001$ (as compared to pre-test)

Subject Retention in Other Longitudinal Studies.

Dr. Comtois and her team review tracking and retention procedures on an ongoing basis to continuously improve these processes. The NIDA R21 pilot study (N=78) of the proposed study had a follow-up rate of 82% at one month. However, our team has been successfully following high-risk populations for outcome assessments for six months or longer for over 22 years. For example, in a naturalistic follow-up of over 200 individuals receiving treatment for self-directed violence in a county emergency department (ED), of whom 61% met criteria for an SUD and 25% were homeless, we achieved an 80% follow-up rate at six months.[32]

Throughout the two latter projects we have used online assessment strategies to obtain questionnaire data and text-based (SMS) technology to reach out to study participants on time. This is accomplished using automated tracking systems embedded in HIPAA-protected and secure [redacted] and REDCap web-based systems. These systems allow the research team to pre-program when assessments or other study activities are due, so the system sends out the correct assessment to the correct study participant at the correct time.

[redacted]

[redacted] If that does not work, the research team is alerted in real time and can use other contact options approved by the participant on our tracking and follow-up consent form to locate the participant, complete that assessment, and obtain the correct information to program back into [redacted] for the next assessment point. [redacted] also has the capacity to track participants without a text-able phone or an email account by sending the alerts to the research team that assessments are due, prompting the team to contact the participant through traditional phone or other means agreed with that participant at baseline.

C2. Overview. This stepped wedge effectiveness trial will randomly assign 15 community treatment settings into five groups of 3 sites each that will be randomly ordered to implement PARS in one of five steps of 6 months each (see Figure 1). The week before the session for PARS in the IOP curriculum within that step, at least 10 clients/site from each site will be consented and complete a baseline assessment. (Standard IOP groups in Washington have 12-15 clients registered and, given 98% of those approached in our pilot study consented, it is expected this will be sufficient.) Participants will be assessed post-intervention and followed up at 1, 3, and 6 months with brief online (or phone, if online or text is not feasible for the participant). Study staff will evaluate counselors at each site to assure fidelity to PARS once trained; supervising as needed throughout the study.

Figure 1 Stepped Wedge Effectiveness Trial Design

	**Step 1**	**Step 2**	**Step 3**	**Step 4**	**Step 5**	**Step 6**
**Group 1 (3 sites)**	TAU	PARS	PARS	PARS	PARS	PARS
**Group 2 (3 sites)**	TAU	TAU	PARS	PARS	PARS	PARS
**Group 3 (3 sites)**	TAU	TAU	TAU	PARS	PARS	PARS
**Group 4 (3 sites)**	TAU	TAU	TAU	TAU	PARS	PARS
**Group 5 (3 sites)**	TAU	TAU	TAU	TAU	TAU	PARS

C3. Design.

Treatment Conditions

Control: Treatment as Usual (TAU)

Intensive Outpatient Program (IOP) guidelines in Washington State are based on State requirements.[33,34] Within these guidelines, agencies typically offer IOP consisting of three-hour groups, three times a week, over eight weeks, for a total of 24 groups (meeting both State and deferred prosecution requirements). Some programs meet less often for a longer time window up to 12 weeks. Although at least one monthly individual counseling session is required, the primary modality is a group format. IOP programs are required to provide education on specific topics, e.g., alcohol and drug education, relapse prevention, risks of drug or alcohol use during pregnancy, blood borne pathogens (including HIV/AIDS and Hepatitis), emotional, physical, and sexual abuse, and nicotine addiction. However, within a 24-group curriculum, IOP programs have wide latitude in determining group content since the required topics typically represent less than 50% of the 24 groups. Therefore the content in IOP programs across our sites is expected to be variable while the structure of session hours and attendance will be consistent. However, no IOP programs include suicide prevention.

At the beginning of the study, each site will select a particular group session in their 24-session schedule to replace with PARS. *They will move that session to the place where PARS will be at the start of the study. To minimize variability between sites, the session to be replaced will be one regarding grief, depression, or coping with negative emotions. While sites vary on what exactly they teach in this general content, all have something they are willing to replace in this non-required section of their IOP, reducing site variability for this study.*

Intervention: TAU + Preventing Addiction Related Suicide (PARS)

PARS is a module designed for a single session of an IOP treatment program including a specified combination of didactic presentations and group discussions. PARS topics include: Goals and Objectives; Suicide Overview; Addiction and Suicide: A Strong Relationship; Suicide Myths and Facts; Suicide Risk Factors; Suicide Protective Factors; Common Triggers of Suicidal Thoughts and Behaviors; Warning Signs and

Guidelines for Preventing Addiction Related Suicide. The PowerPoint slides of the PARS curriculum (and also serving as the training materials) as well as the PARS adherence measure can be found in the Appendix.

The IOP counselor will administer PARS. PARS developer Dr. Ries will provide all training in PARS. In preparation for each site providing PARS, Dr. Ries will provide PARS training using the training model developed in the R21. Dr. Ries and Ms. Kerbrat (Licensed Independent Clinical Social Worker and Research Scientist) will evaluate adherence to PARS by the community treatment counselor in a practice session to assure the counselor can provide PARS with fidelity. Based on the experience from our PARS R21, training will begin one month prior to implementation to assure sufficient time and practice. To maximize the value to our community partners and assure a counselor trained in PARS is available on the day of the PARS module, multiple counselors will participate in the training so that someone can substitute if the designated PARS counselor is unavailable. Dr. Ries and Ms. Kerbrat will assure adherence of all study administration of PARS.

[redacted]

<u>Both Conditions:</u>

Any additional treatments outside of the IOP program (e.g., mental health-oriented counseling, pharmacotherapy) will be available during all phases of the stepped design. Washington State mandates self-help group attendance in addition to attendance of group and individual IOP sessions; this will also remain consistent throughout each site's participation (regardless of when PARS is implemented at each site). Documentation of all services received or referred to will be gathered from the program (with client consent).

Stepped Wedge Design

This study will use a stepped wedge design as shown in Figure 1. This design is recommended for studies in which it is beneficial to randomize at the site rather than individual level.[35–37] Such is the case in this study, as randomization at the individual level would not be feasible with our community partners, who are enrolling clients and placing them in groups based on many complex reasons including client schedules, IOP group schedules and openings, and a myriad of agency guidelines and regulations. Such a study would require conducting PARS and usual care in a research clinic setting—which is the opposite of our goal. PARS was developed in community settings and it is its effectiveness in those settings that is key. Once it was determined that randomization would be at the site level, both clustered randomized and stepped wedge designs were considered.[35,37–44] While both have benefits, the stepped wedge has the advantage of measuring dose-response (delay) effects. Furthermore, its primary disadvantage (that more assessment points were required)[35,40,42] was not determined to be a problem for the research team or community partners, as participants would complete the same assessments regardless of stepped wedge or cluster randomization.

In the proposed study, there will be six steps. Steps will be 6 months each to assure sufficient time for all data collection to occur given that some IOP programs are as long as 12 weeks and sites will vary as to which session PARS replaces in their IOP curricula. In each of the six steps of the study, baseline assessments will be conducted the week before that selected session at that site. For instance, Site A might replace session 8 with PARS but Site B might replace session 14, so baseline assessments will be conducted in week 7 at Site A and week 13 at Site B. (Data collection methods for baseline assessments described in Procedures below.) This will assure that the follow-up assessments reflect the period after PARS is administered while allowing the natural variation in IOP curriculum that is inevitable with 15 community partner sites.

As per the stepped wedge design (see Figure 1), the 15 sites will be randomly assigned to 5 groups of three sites each. At Step 1, all sites will be in the TAU condition. The 5 groups will then be randomly assigned to begin PARS at one of the subsequent 5 steps (i.e., Step 2-6). Once beginning PARS, they will continue to administer PARS through the end of Step 6. Thus, three sites per step will begin administering PARS—until by Step 6, all sites are administering PARS.

Dr. Jim Hughes, stepped wedge design expert (as can be seen in his Biosketch), has reviewed and approved our design (as noted in his Letter of Support) and is available for consultation as needed.

C4. Participants.

Setting. Participants (N=900) will be recruited from 15 community treatment sites in Washington State from the four treatment agencies that are our community partners in this study. As can be seen from their Letters of Support, our community partners are enthusiastic about PARS and see both the program and the study design

as feasible and acceptable. Sites represent urban and rural areas in Washington as well as representing sites primarily funded by private insurance and self-pay and those primarily paid by Medicaid.

Inclusion and Exclusion Criteria

Inclusion Criteria

1. Enrolled client in one of the community treatment settings
2. Over 18 years of age
3. Ability to understand written and spoken English

Exclusion Criteria

1. Any clinical medical/psychiatric condition, severity of that condition, or life situation that in the opinion of the counselors or Drs. Comtois or Ries would compromise safe and voluntary study participation (e.g., psychosis, custody conflict).

Recent suicidal behavior or suicidal ideation is not an exclusion criterion in this study. (While it was in our pilot study, no participant was excluded on this basis.) The scope of chemical dependency counselor's practice in Washington State is to screen and refer acute suicidality to other professionals – often this means outside of the IOP program if a licensed mental health counselor or psychiatric provider is not available. Suicidality is not a static phenomenon – it waxes and wanes for the suicidal individual depending on their internal state and external circumstances. In addition, many individuals do not disclose their suicidality or their suicidality is not acute in while in IOP although it may have been before or after. These individuals are standardly part of IOP programs and they are part of the mission of PARS as are those who have never been suicidal but are at high risk by virtue of substance abuse history severe enough to be in treatment. Thus, all addiction clients regardless of suicidal thoughts or attempt history will be included in the study if they are in treatment at the time of recruitment. We have reviewed with our agency partners the expected incidence of acute suicide risk during a given IOP step. They report this is a rare event. Out of over 200 clients in 15 sites receiving IOP at any given time, they estimate that that 1-3 clients (~1%)are acutely suicidal and may have been referred out.

C5. Recruitment and Consent

The sites in this study vary from 1-3 IOP groups running simultaneously. Therefore, based on a consensus of the research team and community agency for that site, one IOP group in each site will be identified as the best fit for this study based on likely longevity of the counselor over the course of the study and engagement of that counselor with the study and interest in learning PARS. If this identifies more than one group, the decision will include the group with maximum attendance. As noted above, all counselors at the site will be trained in PARS (standard PARS training model) and multiple counselors will be trained to fidelity to assure PARS is administered on schedule to the identified group. Counselors may or may not administer PARS in the other IOP groups as decided by that site. This will not impact the study treatment.

Potential client eligibility will be determined by their IOP counselors. All clients will be asked at the end of a standard treatment group in their IOP if they would like to volunteer for a study that involves staying after that group (or participating before or after one of the other IOP groups that week, if that day is not convenient). If interested, the study will be described to them by the research staff. Interested individuals will be asked to sign the consent form and then complete the baseline assessment.

C.5. Measures

Primary outcome assessments will include knowledge and attitudes toward suicide, and participants' help-seeking behavior for themselves and their family and friends. Exploratory outcomes will be suicidal ideation, threats, and behavior as measured by the Suicidal Behavior Questionnaire - Revised. Potential moderators will also be assessed including measures of drug and alcohol use, depression, physical and mental health functioning, and demographic characteristics. To promote compliance with follow-ups, outcome measures will be delivered via brief, online questionnaires or text message surveys with an option to complete questions via telephone call if participants prefer this. To keep the follow-up measures short, we selected a subset of items used in our initial pilot study that demonstrated optimal psychometric properties. (See Appendix for copies of non-standard measures)

Accurate Information about suicide. The PARS Suicide Knowledge Scale,[1] which was adapted from the Staff Suicide Prevention Survey[45] in our previous pilot trial, was condensed to 6 well-performing items that closely map onto the content of PARS. The Knowledge scale assesses factual understanding of warning signs,

triggers, and interventions for suicide. Items include "Which of the following are warning signs that a person may be thinking about suicide (Mark ALL that apply): a) giving away prized possessions, b) getting a new job, c) talking about death, d) a drug or alcohol relapse, e) increased agitation or anxiety?" Reliability for this shortened scale was good to excellent (Kuder-Richardson 20 = .79 to .99) in the pilot data.

Attitudes toward suicide. The PARS Attitude Scale,[1] originally adapted from the 14-item Staff Suicide Prevention Survey,[45] is rated on a Likert-type scale from 1 (Strongly Disagree) to 5 (Strongly Agree). This scale evaluates stigma and bias toward suicidal acts or persons, as well as perceptions that suicide is preventable if appropriate action is taken. For the proposed study, we selected a subset of seven items using the R21 pilot data to guide item selection. The seven selected items demonstrated acceptable to excellent reliability (α = .73 to .97) and sensitivity to change following PARS program (*ps* < .05) in the pilot study. Sample items include "If someone wants to kill themselves, there is not much anyone can do about it," "Asking a person whether they are feeling suicidal might cause them to do it," and "People who feel suicidal definitely want to die."

**Measure**	**Baseline**	**Post Treatment**	**1, 3, and 6 month Follow-up**
PARS Knowledge Scale	X	X	X
PARS Attitude Scale	X	X	X
PARS Behavior Scale	X		X
Self-harm Behavior Questionnaire	X		
Suicidal Behavior Questionnaire - Revised			X
ASSIST (drug use and alcohol problems)	X		
AUDIT (alcohol use and problems)	X		
Days of drug/alcohol use in past 30 days			X
PHQ-2 (depression)	X		
SF-12 (physical and mental health function)	X		
Demographics	X		
PARS Counselor Acceptability Scale		X	
PARS IOP Discharge Ratings			X

Help-seeking behavior. The PARS Behavior Scale – Client Version[1,26] consists of four items assessing help-seeking behavior for self and others. Participants report the frequency of help-seeking behavior from never ("0 times/none") to "more than 3 times." Items include: "In the past month, have you: (1)… asked a friend to get help because you were worried that he or she was having suicidal thoughts/feelings; (2)… asked a family member or relative to get help because you were worried he or she was having suicidal thoughts/feelings; (3)… asked for help because you were having suicidal thoughts/feelings; and (4)… called a crisis line/suicide hotline?" If items 1 to 3 are endorsed, participants will also be asked to report whom they asked for help. This scale had good to excellent internal consistency in the R21 study (α = .83 to .94) and items were sensitive to the effect of the PARS program. [redacted]

Suicidal ideation and behavior. [redacted] we will use the shorter Suicidal Behaviors Questionnaire-Revised, a four-item self-report that assesses suicide attempts, ideation, communication, and intent since the last assessment. (Time frame will be adapted for each assessment point to gather data for the

time window since the previous assessment.) The SBQ-R has excellent internal consistency (α = .83), sensitivity and specificity (.80 and .91, respectively), and convergence with the SHBQ (r = .77, p < .01).[46,47]

Drug and alcohol use. To assess baseline substance use problems, participants will complete the Alcohol, Smoking and Substance Involvement Screening Test (ASSIST), [48] an 8-item screening tool developed by the World Health Organization to examine lifetime and recent (past three months) substance use, problems resulting from substance use, and risk of current or future harm from substance use. The ASSIST demonstrates acceptable to excellent test-retest reliability (r = .58 to .90), rater agreement (kappas = .61 to .78) and acceptability in substance users. To assess alcohol use problems at baseline, participants will also complete the Alcohol Use Disorders Identification Test (AUDIT) self-report,[49] a 10-item questionnaire that examines frequency, severity, and negative consequences of alcohol use. The AUDIT has demonstrated excellent internal consistency (α = .80 to .98), sensitivity (.74 to .92) and specificity (.71 to .94) in detecting hazardous drinking and alcohol related problems (e.g., hospital admissions, social problems, outpatient treatment, medical disorders).[49–51] At each follow-up, participants will be asked to report on how many of the past 30 days they used drugs or alcohol.

Depression, Physical and Mental Health. Two screening measures will be used to assess covariates and potential moderators (depression, physical health, mental health) of PARS outcomes. The two-item Patient Health Questionnaire (PHQ-2[52–54]) assesses recent depressive symptoms, and has excellent sensitivity (.77 to .86) and specificity (.78 to .95) in detecting major depression.[52,53] The Short-Form 12 Health Survey (SF-12) consists of 12 items assessing overall physical and mental health, and demonstrates excellent test-retest reliability over two weeks (r = .76 to .89) and convergence with other health measures.[55]

Treatment engagement will be measured by a combination of three metrics (a) % sessions attended, (b) graduated from program or terminated, and (c) clinician rating of participant's engagement in IOP treatment. Discharge plan for each client will be determined by a query of the IOP medical record as well as clinician report. (See PARS IOP Discharge Ratings in Appendix).

PARS Acceptability. Addictions counselors will complete the PARS Counselor Acceptability Scale,[1] a 13-item survey that was developed in our R21 trial to measure acceptability, ease, and perceived effectiveness of incorporating PARS into day-to-day IOP procedures where it had excellent internal consistency (α = .99).

C.6. Procedures

Baseline Assessment will be conducted by in-person group administration as described in Recruitment above. If the individual does not have time or requests an individual assessment, this will be offered at a later time or in a different room at the same time. Assessments will be completed on tablet computers.

Post-Intervention Assessment will be conducted after the PARS or TAU group session and include just the information and attitudes items as too little time has passed for other behavioral measures to be relevant.

Follow-Up Assessments will be conducted 1, 3, and 6 months after baseline assessment. Assessments will be completed using online assessments using their smart phone, text message responses, or a computer. If a participant prefers or has any technical problems, follow-up assessments can be conducted by phone. Dr. Comtois' current trials use these modalities to complete questionnaire follow-up assessments with over 500 Soldiers and Marines. Online methods are extremely effective with a very mobile population such as Service Members who move while active duty and as they separate from the military and also for those in addiction treatment who are often moving residences or homeless.

In Drs. Comtois' and Ries' ongoing trial of caring contacts via text message with suicidal active duty military personnel, we are utilizing a text messaging platform designed and maintained by [redacted] that is capable of administering surveys via text message. We have demonstrated through extensive testing and implementation that the system is fully capable of obtaining follow-up survey responses via text message. The [redacted] system records every text "question" sent by the system reliably as a success or failure (i.e., the SMS "succeeded" in delivery to the participants' mobile phone, or the SMS "failed" to go through). This function is highly reliable and our team is well-practiced in addressing any issues with undeliverable text messages. Our group uses Research Electronic Data Capture (REDCap) online surveys to reliably collect online survey data via any device with an internet connection (e.g., smartphone, tablet, desktop computer). As described in the Human Subjects section, both proposed data collection systems are HIPAA compliant and designed for clinical and research applications.[56] (See Letter of Support from [redacted] team.)

Steps to Prevent Participant Attrition. Our goal is for all participants to complete all outcome assessments regardless of treatment participation. Informed consent will include a separate tracking consent form on which participants choose tracking strategies they consent to have used (e.g., obtaining forwarding address from post office, checking social media) and provide alternative contacts to whom the study can reach out in case the

participant moves or changes contact information. We have used this tracking consent form for over 20 years of research. It finds an excellent balance between obtaining detailed information and allowing participants to only provide information they are comfortable providing. *In our previous studies, we have achieved 80% follow-up in the six months following an ER admission for self-injury in a past study[32] and an 82% six month follow-up rate for our current study of suicidal Marines and Soldiers. In addition, we have conducted a literature review of methods to achieve high subject retention in substance abuse studies.[57–59] While we already use most of these procedures, we will follow all the recommendations to minimize attrition in this study.*

Participants will be reimbursed (in a choice of gift cards) $30 for their time for each baseline, $20 for post-treatment, and follow-up assessments in increments of $10 for each 10 questions completed. To minimize attrition, participants will also be offered an addition incentive to be paid at the final 6-month assessment (or end of the 6-month assessment window if they do not complete it). The additional incentive (also in gift cards) will be $20 for completing 2 of the 3 outcome assessments and $30 for completing all outcome assessments.

C.7. Data Analytic Plan

Data screening and preliminary analyses. First, exploratory data analysis will be performed to characterize the distributional characteristics of all variables. Frequency distributions and plots will be examined to identify out-of-range values and to assess data distributions. To assess scale reliability and validity, item structure will be examined using Cronbach's alpha and factor analysis. Descriptive statistics, stratified by site and study condition, will be examined to characterize sample characteristics and assess randomization. Baseline characteristics including socio-economic status, gender, suicidality, or substance use found to vary significantly between study conditions will be included as covariates in subsequent outcome analyses. Plots of mean outcomes by assessment point, site, and study condition will be examined to characterize outcome trajectories over time and inform the parameterization of time in the longitudinal models. Because differential attrition can compromise inference about experimental effects, we will compare dropouts and completers on demographic characteristics and available outcome variables.

Primary outcome analyses**.** In the proposed stepped wedge design, 15 addiction treatment sites that initially provide TAU (Step 1) will be randomized to implement PARS beginning at one of five ensuing steps (Steps 2-6), 3 sites at a time. Participants recruited at each step will be assessed at points: baseline, post-treatment (information and attitudes only), 1, 3, and 6 months. The primary outcome analyses will be intent-to-treat analyses that include all participants, including those with incomplete follow-up data. The primary outcomes will be 1) knowledge about suicide, 2) maladaptive attitudes towards suicide, and 3) help-seeking behaviors. Each outcome will be evaluated in a separate generalized linear mixed model (GLMM)[60] to evaluate the effect of PARS program vs. Treatment As Usual on change in each outcome over time. The GLMM approach accounts for within-individual and between-group variance and is well-suited for the analyses of multisite, repeated measures data. A logit link function will be used to model dichotomous outcomes and a Gaussian link function when the outcome is relatively normally distributed.

The following regression equation depicts the basic model for each outcome: $OUTCOME_{tis} = b_0 + b_1STEP2_{is} + b_2STEP3_{is} + b_3STEP4_{is} + b_4STEP5_{is} + b_5STEP6_{is} + b_6SETTING_{is} + b_7PARS_{is} + b_8MONTH1_{tis} + b_9MONTH3_{tis} + b_{10}MONTH6_{tis} + b_{11}(PARS_{is} * MONTH1_{tis}) + b_{12}(PARS_{is} * MONTH3_{tis}) + b_{13}(PARS_{is} * MONTH6_{tis}) + u_{0s} + r_{0is} + e_{tis}$, *where t indexes the assessment, i indexes the individual, and s indexes the site. In this model, each outcome is regressed on Step, Setting (Rural vs. Urban), Treatment (PARS vs. TAU), Time (in months), and the Treatment by Time interaction. The effect of Step will be coded into contrasts of Step 1 against each subsequent step (i.e., Step 2 vs 1, Step 3 vs 1, etc.). The effect of Time will be coded into contrasts of baseline (BL) against each post-baseline assessment (i.e., BL vs. 1 month, BL vs. 3, BL vs. 6 months). To account for the cluster-randomized design, a random effect for site* (u_{0s}) *will be included to model the correlation of individuals from the same site. To account for the repeated assessments, a random effect for participant* (r_{0is}) *will be included to model the correlation of observations within the same individual. The statistical test of the treatment effect will be the magnitude and statistical significance of the Treatment by Time interactions. Comparable analyses will be use for exploratory aims with suicidality and substance use.*

Since this study will be carried out at 15 sites, there is the potential for site-specific effects. We anticipate intervention effects to be comparable across all clinics since they are all serving similar client populations and have counselors with comparable levels of experience and training. To verify that treatment effects are comparable across site, preliminary outcome analyses will incorporate indicator variables for site and their interactions with the treatment effects. A likelihood ratio test will be used to compare the models with and without site-specific effects. If the likelihood ratio tests reveal differences in treatment effect by site, these

site-specific parameters will be retained in the final analyses, otherwise they will be excluded.

Secondary Analyses

Moderation Analyses. We will examine whether individual-level factors (e.g., demographic characteristics, drug and alcohol use, depression, physical and mental health functioning, treatment engagement) moderate the effect of the PARS program. To evaluate these individual-level moderators, the primary outcome analyses above will be extended to include the main effect of the moderator and its interactions with Treatment, Time, and the Treatment by Time interactions. Each moderator will first be evaluated in a separate model to ascertain whether any differences in treatment effects were associated with individual level factors. A follow-up model will evaluate the moderators simultaneously in a single model. The statistical test of moderation will be the three-way Treatment by Time by Moderator interactions.

Mediation Analyses. Secondary mediation analyses will examine whether baseline to post-intervention 1) increases in knowledge about suicide and 2) decreases in maladaptive attitudes about suicide will mediate the effect of PARS vs. TAU help-seeking behaviors. The mediation analyses will be conducted using multilevel structural equation modeling.[61] Robust standard error estimates and corrected model fit statistics (e.g., Satorra-Bentler statistic) will be utilized to accommodate non-normally-distributed dependent variables in the model.[62] Each mediator will first be evaluated in a separate model. Figure 2 illustrates the basic autoregressive regression model[63] that will be used to assess longitudinal mediation of the effect of intervention on 1-, 3-, and 6-month help-seeking behavior. An indicator variable for Step will be included as a covariate if the effect of step is statistically significant in the primary outcome analyses. The model will incorporate random effects for site and individual to account for the clustered design (i.e., repeated measures nested within individuals, nested within site). The mediated effect (a_1*b_5) and its confidence interval will be obtained using bootstrap resampling, as recommended for testing indirect effects.[64] (Note, arrows on upper right side of mediator and outcome boxes represent error terms.)

Figure 2

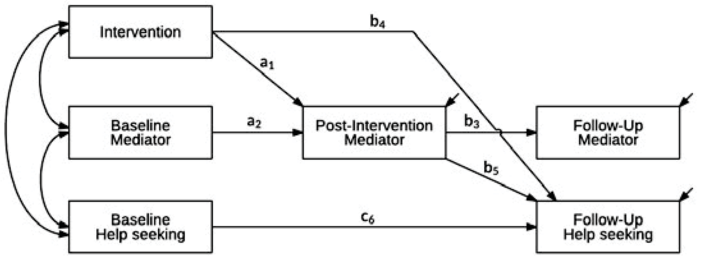

Dose Response Analyses. Exploratory analyses will evaluate whether Time (i.e., number of steps) in the intervention phase is associated with improved outcomes in successive steps. The following regression equation depicts the basic dose response model for each outcome: $OUTCOME_{t>0,is} = b_0 + b_1 BL\_OUTCOME_{is} + b_2 TAU\_TIME_{is} + b_3 PARS\_TIME_{is} + u_{0s} + r_{0is} + e_{tis}$, where t indexes the assessment, i indexes the individual, and s indexes the site. In this model, Time will be centered at the beginning of the intervention phase, such that Time = 0 will correspond to the step in which PARS was implemented at a particular site. Time will be coded as a piecewise linear function representing two phases: 1) the TAU phase: the change over time in outcomes during the control period and the 2) PARS phase: a “deflection” term representing the change in slope from the TAU phase following the implementation of PARS. To account for the cluster-randomized design, a random effect for site (u_{0s}) will be included to model the correlation of individuals from the same site. To account for the repeated assessments, a random effect for participant (r_{0is}) will be included to model the correlation of observations within the same individual. The statistical test of the dose-response effect will be the magnitude and statistical significance of the slope of Time during the intervention period.

Power Analysis**.** We base our power analysis on a stepped wedge design[35] evaluating the difference in the rate of help-seeking behavior at month 1 (post-intervention) among those receiving PARS vs. TAU. Based on our pilot study of PARS, we anticipate a 15.6% baseline rate of help seeking behavior, which we expect to increase to 28.1% at post-intervention (1-month) follow-up. Based on the proposed stepped wedge design in which 10 individuals are recruited per site in each of the 6 steps (*N* = 900), we will have 89% power to detect the 12.5% difference in the rate of help-seeking behavior. Assuming 25% fewer participants (*N* = 675, 7.5 individuals per site per step), due to under-recruitment and/or attrition, there will still be 80% power to detect an intervention effect. This represents a conservative estimate of statistical power as the full longitudinal model will leverage data from all participants, including those with incomplete follow-up data.

Missing data. Missing data may occur in several ways. First, missing data may occur due to item non-response. When missing data is limited to only a few items on a measure, we will prorate total scores for a measure by taking an average score on the measure and multiplying it by the total number of items in the scale. Missing data can also occur from attrition due to missed assessments or dropout from the study. Prior to performing any outcome analyses, we will evaluate the amount, reasons, and patterns of missing data. If the

reason for missing data is not related to the outcome of interest, then the missing data are considered to be missing completely at random and complete case analysis will still generate unbiased estimates.[65] A multiple imputation using chained equations approach will be utilized to address missing data, with the final results calculated as a pooled average across 10 multiply-imputed data sets using Rubin's methodology.[66] We will conduct sensitivity analyses to compare estimates of treatment effects with and without multiple imputation to assess the effect of missing data on statistical inference.

C.8 Dissemination Plan

Dr. Ries will lead a PARS Advisory Group as he did in the R21 study including Dr. Comtois and Ms. Kerbrat as well as Dr. Dennis Donovan, and three agency heads: [redacted] (see Letters of Support for their willingness to participate). The Advisory Group will meet periodically throughout the study and then will have multiple meetings in Year 5 to develop a dissemination plan if PARS is effective.

C.9. Time Line

	Year 1		Year 2		Year 3		Year 4		Year 5	
	0-6	7-12	0-6	7-12	0-6	7-12	0-6	7-12	0-6	7-12
Hire and train personnel	■									
Organize study with partners	■									
Step 1 (baseline for all sites)		■								
Step 2 (3 new sites start PARS)			■							
Step 3 (3 new sites start PARS)				■						
Step 4 (3 new sites start PARS)					■					
Step 5 (3 new sites start PARS)						■				
Step 6 (3 new sites start PARS)							■			
Follow-up assessments		■	■	■	■	■	■	■		
Conduct outcome analysis								■	■	■
Disseminate study results									■	■

C.10. Study Limitations

- We have selected only one IOP group per site to participate in the study. We decided on this approach as some sites only have one IOP group, but it does potentially limit our sample size. However, as noted in the Power Analysis section (Section C.7), we have sufficient power even with a smaller sample size as we have increased power with both the number of sites and number of steps. We will select the most stable and strongest group at each site. While this can lead to selection bias, we believe it will increase internal validity to have as strong a PARS condition as possible with a maximally motivated and stable staff. In the first controlled trial, it is important that PARS be as strong as possible to prevent abandoning a potentially effective program. Future research can determine the range of clinician motivation and skill associated with effective PARS administration.
- Our outcome assessment instruments are all brief. While this is a limitation, we have selected psychometrically strong measures *and collect clinician collateral data*. And with a sample of 900 participants, it is critical to collect as much data as possible using text message and online surveys, which require brief measures. Finally, to minimize attrition it will be critical to minimize the assessment burden.
- For our information, attitude, and help-seeking measures, we have selected the measures developed for the R21 study rather than existing measures. After a detailed review of the literature, many measures were found to be very long and repetitive, contain outdated suicide information, or both. The measures included in this study were selected because they are based on existing measures, have accurate items, show strong internal consistency in pilot work, have content validity with PARS curriculum, and are brief.
- One possible limitation to our outcome assessment protocol is the potential priming of effect by repeated assessment. However, we believe the precision will be increased and repeated assessment is necessary for mediation and dose analyses. The effect will be equated between conditions through randomization.
- Given that many clients in addiction treatment are under acute stress, drop out, and are in unstable living situations, attrition is a significant risk. Several strategies have been included to overcome this limitation including a post-intervention assessment, using established tracking strategies *based on a review of substance abuse longitudinal research recommendations*, obtaining multiple follow-up contacts, brief assessments that require little time and coordination, and subject reimbursement incentives.

References

1. Voss, W. D. *et al.* Preventing addiction related suicide: A pilot study. *J. Subst. Abuse Treat.* 44, 565–569 (2013).
2. Yuodelis-Flores, C. & Ries, R. K. Addiction and suicide: A review. *Am. J. Addict. Am. Acad. Psychiatr. Alcohol. Addict.* 24, 98–104 (2015).
3. Wilcox, H. C., Conner, K. R. & Caine, E. D. Association of alcohol and drug use disorders and completed suicide: an empirical review of cohort studies. *Drug Alcohol Depend.* 76, Supplement, S11–S19 (2004).
4. Darke, S. & Ross, J. The relationship between suicide and heroin overdose among methadone maintenance patients in Sydney, Australia. *Addiction* 96, 1443–1453 (2001).
5. Wojnar, M. *et al.* Impulsive and non-impulsive suicide attempts in patients treated for alcohol dependence. *J. Affect. Disord.* 115, 131–139 (2009).
6. Kessler, R. C., Borges, G. & Walters, E. E. Prevalence of and risk factors for lifetime suicide attempts in the national comorbidity survey. *Arch. Gen. Psychiatry* 56, 617–626 (1999).
7. Preuss, U. W. *et al.* Predictors and correlates of suicide attempts over 5 years in 1,237 alcohol-dependent men and women. *Am. J. Psychiatry* 160, 56–63 (2003).
8. United States, Public Health Service, Office of the Surgeon General & National Action Alliance for Suicide Prevention (U.S.). *2012 national strategy for suicide prevention: goals and objectives for action : a report of the U.S. Surgeon General and of the National Action Alliance for Suicide Prevention.* (2012). at <http://www.ncbi.nlm.nih.gov/books/NBK109917/>
9. Rounsaville, B. J., Carroll, K. M. & Onken, L. S. A Stage Model of Behavioral Therapies Research: Getting Started and Moving on From Stage I. *Clin. Psychol. Sci. Pract.* 8, 133–142 (2001).
10. Carroll, K. M. & Onken, L. S. Behavioral Therapies for Drug Abuse. *Am. J. Psychiatry* 162, 1452–1460 (2005).
11. SAMHSA/CSAT. *TIP 50: Addressing Suicidal Thoughts and Behaviors in Substance Abuse Treatment - Literature Review with Update*. (2012). at <http://store.samhsa.gov/product/TIP-50-Addressing-Suicidal-Thoughts-and-Behaviors-in-Substance-Abuse-Treatment/SMA09-4381>

12. Center for Substance Abuse Treatment. *Substance Abuse Treatment: Group Therapy*. (Substance Abuse and Mental Health Services Administration (US), 2005). at <http://www.ncbi.nlm.nih.gov/books/NBK64220/>
13. American Foundation For Suicide Prevention. Facts and Figures. (2015). at <www.afsp.org/understanding-suicide/facts-and-figures>
14. CDC - Injury - WISQARS Cost of Injury Reports. at <http://wisqars.cdc.gov:8080/costT/>
15. Ross, J. *et al.* The characteristics of heroin users entering treatment: findings from the Australian treatment outcome study (ATOS). *Drug Alcohol Rev.* 24, 411–418 (2005).
16. Kwon, M., Yang, S., Park, K. & Kim, D.-J. Factors that affect substance users' suicidal behavior: a view from the Addiction Severity Index in Korea. *Ann. Gen. Psychiatry* 12, 35 (2013).
17. Manning, V. *et al.* Suicidal ideation and lifetime attempts in substance and gambling disorders. *Psychiatry Res.* 225, 706–709 (2015).
18. Tiet, Q. Q., Ilgen, M. A., Byrnes, H. F. & Moos, R. H. Suicide Attempts Among Substance Use Disorder Patients: An Initial Step Toward a Decision Tree for Suicide Management. *Alcohol. Clin. Exp. Res.* 30, 998–1005 (2006).
19. Ortíz-Gómez, L. D., López-Canul, B. & Arankowsky-Sandoval, G. Factors associated with depression and suicide attempts in patients undergoing rehabilitation for substance abuse. *J. Affect. Disord.* 169, 10–14 (2014).
20. Rudd, M. D. Fluid Vulnerability Theory: A Cognitive Approach to Understanding the Process of Acute and Chronic Suicide Risk. doi:10.1037/11377-016
21. Kirchberg, T. M. & Neimeyer, R. A. Reactions of beginning counselors to situations involving death and dying. *Death Stud.* 15, 603–610 (1991).
22. Ross, J., Darke, S., Kelly, E. & Hetherington, K. Suicide risk assessment practices: a national survey of generalist drug and alcohol residential rehabilitation services. *Drug Alcohol Rev.* 31, 790–796 (2012).
23. Conner, K. R., Wood, J., Pisani, A. R. & Kemp, J. Evaluation of a suicide prevention training curriculum for substance abuse treatment providers based on Treatment Improvement Protocol Number 50. *J. Subst. Abuse Treat.* 44, 13–16 (2013).

24. Orwall, T. *Washington State Matt Adler Suicide Assessment, Management, and Treatment Act*. at <http://apps.leg.wa.gov/billinfo/summary.aspx?bill=2366&year=2011>
25. Morley, K. C., Sitharthan, G., Haber, P. S., Tucker, P. & Sitharthan, T. The efficacy of an opportunistic cognitive behavioral intervention package (OCB) on substance use and comorbid suicide risk: a multisite randomized controlled trial. *J. Consult. Clin. Psychol.* 82, 130–140 (2014).
26. Aseltine, R. H. & DeMartino, R. An Outcome Evaluation of the SOS Suicide Prevention Program. *Am. J. Public Health* 94, 446–451 (2004).
27. Aseltine, R. H., James, A., Schilling, E. A. & Glanovsky, J. Evaluating the SOS suicide prevention program: a replication and extension. *BMC Public Health* 7, 161 (2007).
28. Eggert, L., Thompson, E., Randell, B. & Pike, K. Preliminary effects of brief school-based prevention approaches for reducing youth suicide -- risk behaviors, depression, and drug involvement. *J. Child Adolesc. Psychiatr. Nurs.* 15, 48–64 (2002).
29. Thompson, E. A., Eggert, L. L., Randell, B. P. & Pike, K. C. Evaluation of indicated suicide risk prevention approaches for potential high school dropouts. *Am. J. Public Health* 91, 742–752 (2001).
30. Randell, B. P., Eggert, L. L. & Pike, K. C. Immediate post intervention effects of two brief youth suicide prevention interventions. *Suicide Life. Threat. Behav.* 31, 41–61 (2001).
31. Stenbacka, M., Leifman, A. & Romelsjö, A. Mortality and cause of death among 1705 illicit drug users: A 37 year follow up. *Drug Alcohol Rev.* 29, 21–27 (2010).
32. Comtois, K. A., Kerbrat, A. H. M., Atkins, D. C., Roy-Byrne, P. & Katon, W. Self-reported Usual Care for Self-directed Violence During the 6 Months Before Emergency Department Admission. *Med. Care* 53, 45–53 (2015).
33. WAC 388-877B-0300. *Chemical dependency outpatient treatment services—General. Washington Administrative Code* 388-877B-0300, (2013).
34. WAC 388-877B-0350. *Chemical dependency outpatient treatment services requiring program-specific certification - Level II intensive outpatient services*. *Washington Administrative Code* 388-877B0350, (2013).
35. Hussey, M. A. & Hughes, J. P. Design and analysis of stepped wedge cluster randomized trials. *Contemp. Clin. Trials* 28, 182–191 (2007).

36. Sareen, J. *et al.* Promising Strategies for Advancement in Knowledge of Suicide Risk Factors and Prevention. *Am. J. Prev. Med.* 47, S257–S263 (2014).
37. Mdege, N. D., Man, M.-S., Taylor (nee Brown), C. A. & Torgerson, D. J. Systematic review of stepped wedge cluster randomized trials shows that design is particularly used to evaluate interventions during routine implementation. *J. Clin. Epidemiol.* 64, 936–948 (2011).
38. Dreischulte, T. *et al.* A cluster randomised stepped wedge trial to evaluate the effectiveness of a multifaceted information technology-based intervention in reducing high-risk prescribing of non-steroidal anti-inflammatory drugs and antiplatelets in primary medical care: The DQIP study protocol. *Implement. Sci.* 7, 24 (2012).
39. Kotz, D., Spigt, M., Arts, I. C. W., Crutzen, R. & Viechtbauer, W. Researchers should convince policy makers to perform a classic cluster randomized controlled trial instead of a stepped wedge design when an intervention is rolled out. *J. Clin. Epidemiol.* 65, 1255–1256 (2012).
40. Woertman, W. *et al.* Stepped wedge designs could reduce the required sample size in cluster randomized trials. *J. Clin. Epidemiol.* 66, 752–758 (2013).
41. Zhan, Z. *et al.* Strengths and weaknesses of a stepped wedge cluster randomized design: its application in a colorectal cancer follow-up study. *J. Clin. Epidemiol.* 67, 454–461 (2014).
42. Mdege, N. D., Man, M.-S., Taylor (nee Brown), C. A. & Torgerson, D. J. There are some circumstances where the stepped-wedge cluster randomized trial is preferable to the alternative: no randomized trial at all. Response to the commentary by Kotz and colleagues. *J. Clin. Epidemiol.* 65, 1253–1254 (2012).
43. Kotz, D., Spigt, M., Arts, I. C. W., Crutzen, R. & Viechtbauer, W. Use of the stepped wedge design cannot be recommended: A critical appraisal and comparison with the classic cluster randomized controlled trial design. *J. Clin. Epidemiol.* 65, 1249–1252 (2012).
44. Hill, A.-M. *et al.* A stepped-wedge cluster randomised controlled trial for evaluating rates of falls among inpatients in aged care rehabilitation units receiving tailored multimedia education in addition to usual care: a trial protocol. *BMJ Open* 4, e004195 (2014).
45. Wyman, P. A. *et al.* Randomized trial of a gatekeeper program for suicide prevention: 1-year impact on secondary school staff. *J. Consult. Clin. Psychol.* 76, 104–115 (2008).

46. Gutierrez, P. M., Osman, A., Barrios, F. X. & Kopper, B. A. Development and initial validation of the Self-harm Behavior Questionnaire. *J. Pers. Assess.* 77, 475–490 (2001).

47. Osman, A. *et al.* The Suicidal Behaviors Questionnaire-Revised (SBQ-R): validation with clinical and nonclinical samples. *Assessment* 8, 443–454 (2001).

48. Group, W. A. W. The Alcohol, Smoking and Substance Involvement Screening Test (ASSIST): development, reliability and feasibility. *Addiction* 97, 1183–1194 (2002).

49. Saunders, J. B., Aasland, O. G., Babor, T. F., de la Fuente, J. R. & Grant, M. Development of the Alcohol Use Disorders Identification Test (AUDIT): WHO Collaborative Project on Early Detection of Persons with Harmful Alcohol Consumption--II. *Addict. Abingdon Engl.* 88, 791–804 (1993).

50. Conigrave, K. M., Saunders, J. B. & Reznik, R. B. Predictive capacity of the AUDIT questionnaire for alcohol-related harm. *Addiction* 90, 1479–1485 (1995).

51. Conigrave, K. M., Hall, W. D. & Saunders, J. B. The AUDIT questionnaire: choosing a cut-off score. *Addiction* 90, 1349–1356 (1995).

52. Arroll, B. *et al.* Validation of PHQ-2 and PHQ-9 to screen for major depression in the primary care population. *Ann. Fam. Med.* 8, 348–353 (2010).

53. Inagaki, M. *et al.* Validity of the Patient Health Questionnaire (PHQ)-9 and PHQ-2 in general internal medicine primary care at a Japanese rural hospital: a cross-sectional study. *Gen. Hosp. Psychiatry* 35, 592–597 (2013).

54. McManus, D., Pipkin, S. S. & Whooley, M. A. Screening for depression in patients with coronary heart disease (data from the Heart and Soul Study). *Am. J. Cardiol.* 96, 1076–1081 (2005).

55. Ware, J., Jr, Kosinski, M. & Keller, S. D. A 12-Item Short-Form Health Survey: construction of scales and preliminary tests of reliability and validity. *Med. Care* 34, 220–233 (1996).

56. Harris, P. A. *et al.* Research electronic data capture (REDCap)--a metadata-driven methodology and workflow process for providing translational research informatics support. *J. Biomed. Inform.* 42, 377–381 (2009).

57. Walton, M. A., Ramanathan, C. S. & Reischl, T. M. Tracking Substance Abusers in Longitudinal Research: Understanding Follow-Up Contact Difficulty. *Am. J. Community Psychol.* 26, 233–253 (1998).

58. Kleschinsky, J. H., Bosworth, L. B., Nelson, S. E., Walsh, E. K. & Shaffer, H. J. Persistence pays off: follow-up methods for difficult-to-track longitudinal samples. *J. Stud. Alcohol Drugs* 70, 751–761 (2009).
59. Scott, C. K. A replicable model for achieving over 90% follow-up rates in longitudinal studies of substance abusers. *Drug Alcohol Depend.* 74, 21–36 (2004).
60. Diggle, P., Heagerty, P., Liang, K.-Y. & Zeger, S. *Analysis of Longitudinal Data*. (Oxford Univeristy, 2013).
61. Hox, J. *Multilevel analysis: Techniques and applications*. (Routledge, 2010).
62. Kline, R. B. *Principles and Practice of Structural Equation Modeling*. (The Guilford Press, 2010).
63. MacKinnon, D. P. *Introduction to Statistical Mediation Analysis*. (Routledge, 2008).
64. Shrout, P. E. & Bolger, N. Mediation in experimental and nonexperimental studies: New procedures and recommendations. *Psychol. Methods* 7, 422–445 (2002).
65. Rubin, D. B. *Multiple Imputation for Nonresponse in Surveys*. (Wiley-Interscience, 2004).
66. Little, R. J. A. & Rubin, D. B. *Statistical Analysis with Missing Data*. (John Wiley & Sons, Ltd, 1987).
67. Gould, M. S. *et al.* Evaluating iatrogenic risk of youth suicide screening programs: a randomized controlled trial. *JAMA J. Am. Med. Assoc.* 293, 1635–1643 (2005).
