# Supplementary material for: Effectiveness of a Suicide Prevention Module for Adults in Substance Use Disorder Treatment: A Stepped-Wedge Cluster-Randomized Clinical Trial
Source: JAMA Netw Open. 2022 Apr 6;5(4):e222945. doi: 10.1001/jamanetworkopen.2022.2945 (PMC8987906; doi:10.1001/jamanetworkopen.2022.2945)
Supplement: Supplement 2. — Data Sharing Statement. [file jamanetwopen-e222945-s002.pdf]

Data Sharing Statement

Ries. Effectiveness of a Suicide Prevention Module for Adults in Substance Use Disorder Treatment. *JAMA Netw Open*. Published April 06, 2022.
doi:10.1001/jamanetworkopen.2022.2945

Data
Data available: No

Additional Information
Explanation for why data not available: Data sharing was and is not explicitly planned. Per our protocol and informed consent, data sharing is possible if requested under a data sharing agreement. If a researcher requests a data sharing agreement, they would first be required provide the study investigators with a proposal of hypotheses, variables needed to test these hypotheses, and plans for dissemination of findings. As part of the data sharing agreement, researchers requesting data would be required to indicate in a signed document approved by the principal investigator: (1) completion of all required ethics and regulatory training; (2) a commitment to using the data only for research purposes; (3) a plan for securing the data; (4) an agreement to either destroy or return the data once analyses are completed; and (5) an agreement to not share data with other users, and to direct all such requests to the principal investigator. Per our protocol and informed consent, only de-identified data would be shared.
